# Diversity and plant growth-promoting traits of endophytic bacteria isolated from maize cropped in organic and low-input agricultural systems in southern Brazil

**DOI:** 10.1099/acmi.0.000948.v3

**Published:** 2026-06-03

**Authors:** Dafila Santos Lima Fagotti, Josiane Fukami, Paula Cerezini, Andre Luiz Martinez Oliveira, Galdino Andrade, Milena Serenato Klepa, Luisa Carolina Ferraz Helene, Ligia Maria Oliveira Chueire, Renan Augusto Ribeiro, Mariangela Hungria, Marco Antonio Nogueira

**Affiliations:** 1 Department of Agronomy, State University of Londrina, Londrina, PR, Brazil; 2 Department of Biochemistry and Biotechnology, State University of Londrina, Londrina, PR, Brazil; 3 Department of Microbiology, State University of Londrina, Londrina, PR, Brazil; 4 Fellow of the National Council for Science and Technology - CNPq, Brasília, DF, Brazil; 5 Vittia Fertilizantes e Biológicos, São Joaquim da Barra, São Paulo, Brazil; 6 Embrapa Soja, Cx. Postal 4006, 86085-981, Londrina, PR, Brazil

**Keywords:** 16S rRNA gene, bacterial diversity, biocontrol, functional genes, organic farming, plant growth promotion, whole-genome sequencing

## Abstract

Plant growth-promoting bacteria (PGPB) may enhance plant growth and health through several mechanisms, contributing to sustainable agriculture. We isolated and characterized endophytic bacteria from stems of landrace maize (*Zea mays* L.) grown under low-input and organic systems in southern Brazil. The 16S rRNA analysis revealed 83 isolates within 14 genera, including *α*-Proteobacteria, *β-Proteobacteria*, *γ-Proteobacteria*, *Actinomycetia* and *Bacilli* classes. The synthesis of indolic compounds was widespread among the strains, as well as the enhancement of N concentration in N-free media, a putative trait for the capacity of biological nitrogen fixation. Phosphate solubilization prevailed in *Burkholderia* and *Paraburkholderia* strains, while siderophore production was observed in all genera except *Microbacterium*. Inoculation with selected strains confirmed growth promotion on maize plantlets, particularly *Sphingomonas* CNPSo 2378 and *Bacillus velezensis* CNPSo 2384, whose genomes were sequenced and revealed PGPB features and evolutionary relationships, shedding light on the diversity, functional traits and occurrence of PGPB in low-input and organic agricultural systems.

## Data Summary

The 16S rRNA gene sequences have been deposited in the National Center for Biotechnology Information GenBank database, with accession numbers provided in the supplementary material (Table S2). The genomic sequences have also been deposited in the GenBank database, with the following accession numbers: JAXCMJ000000000 for *Sphingomonas* CNPSo 2378 and JAXCMK000000000 for *Bacillus velezensis* CNPSo 2384. Raw data on the effects of bacterial isolates on the plant growth promotion of maize plantlets are available at the figshare database, with the following DOI: 10.6084 /m9.figshare.27610290.

## Introduction

Plant growth-promoting bacteria (PGPB) play a pivotal role in enhancing plant development and yield, making them valuable allies of sustainable agriculture. PGPB are often associated with the rhizosphere, rhizoplane or inhabiting plant tissues endophytically, and may play a role in nutrient uptake, particularly nitrogen, phosphorus and iron, based on processes such as biological nitrogen fixation, P-solubilization and production of siderophores, respectively [1,2]. Additionally, PGPB may stimulate plant growth based on the production of phytohormones such as auxins, cytokinins and gibberellins, which regulate plant growth and development [1,3]. They also may suppress plant pathogens by producing antimicrobial compounds and competing for nutrients and space [4,5]. Moreover, PGPB can improve plant tolerance to environmental stresses such as drought, salinity and heavy metal toxicity by producing protective compounds and inducing systemic resistance [3,6].

Among the main crops favoured by PGPB, maize (*Zea mays* L.) stands out in global agriculture due to its widespread cultivation and vital role in food security. In addition, maize is well-known for its adaptability to diverse environments, harbouring a complex microbiome that includes PGPB, which contribute significantly to crop health and yield [7,9].

The different agricultural cropping systems, whether organic or conventional, with low or high use of inputs, deeply influence the composition and function of microbial communities associated with crops. Organic agriculture emphasizes sustainable practices, minimizing synthetic inputs and prioritizing soil health and biodiversity. In contrast, conventional agriculture often relies on intensive use of inputs, including fertilizers and pesticides. These different management approaches may shape the microbial diversity in agricultural ecosystems, in which organic systems typically foster larger microbial abundance and diversity [10,11]. Despite the agreement that closer plant–micro-organism interactions may occur in organic and low-input cropping systems, there are few studies on the diversity and abundance of endophytic bacteria in maize.

Understanding the microbial communities' composition in organic and low-input conventional agricultural systems is essential for optimizing plant health and yield while minimizing environmental impacts. This study aimed to isolate endophytic bacteria from maize grown under organic and low-input cropping systems and proceed with their taxonomic identification and evaluation of plant growth-promoting traits for possible further use as PGPB in sustainable agricultural systems.

## Methods

### Bacterial isolation and morphological characterization of colonies

Root crowns of landrace maize varieties at the flowering stage, cropped under organic, transition from conventional to organic and low-input conventional systems were sampled for isolation of endophytic bacteria. Samples were taken in the austral summer in five sites ranging from 753 to 807 m a.s.l., in three municipalities of the Parana state, southern Brazil. These regions present a humid subtropical climate – Cfa (Köppen’s classification), with moderate summers and frequent frosts in winter. The average minimum and maximum temperatures throughout the year are −5 and 38 °C. The rain is well distributed in all seasons, with averages ranging from 1,530 to 1,700 mm per year. Details on the sampling sites and results of soil chemical analysis are shown in Table S1 (available in the online Supplementary Material).

In each site, 10 plants at full bloom were randomly chosen, and the crown region (5–8 cm length) was taken, amounting to 50 samples. The material was washed in tap water, surface-disinfected with 70% ethanol (3 min) and 20% commercial hypochlorite (5 min) and then rinsed with sterile water and transferred to a 0.05 mol l^−1^ phosphate buffer. Aiming at selecting diazotrophic endophytes, 10 g of samples was crushed in 90 ml of 0.85% sterile saline, and 50 µl of the supernatant was inoculated in two vials containing 5 ml of N-free semi-solid culture media LGI, JNFb or JMV [12]. After incubation for 7 days at 30 °C in the dark, vials showing typical growth, i.e. alkalinization of the medium and formation of growth-pellicle below the medium surface, were used for isolation following four sequential reinoculations (stabbing) in the same culture medium in new vials to decrease the probability that the growth was due to residual N from the previous inoculated cells or residual N brought from the dilution process of the sample [13]. After the fourth stabbing, the growth was streaked on the respective solidified culture medium to obtain isolated, morphologically different colonies. A total of 83 isolates were obtained based on different colony morphotypes and were further characterized as described in the following steps.

### Genomic DNA extraction, amplification and sequencing of the 16S rRNA gene

The isolates were grown in the dye-glucose-yeast-glumatate (DYGS) medium broth [14] for 24 h and centrifuged. The genomic DNA of the bacterial pellet was extracted with the Bacterial DNA Kit D3350-02 (Omega Bio-Tek, USA) and purified with the PureLink^™^ PCR Purification Kit (Invitrogen, Brazil).

The amplification and sequencing of the 16S rRNA gene were performed as previously described [15]. High-quality sequences obtained for each strain were assembled into contigs using the software Phred [16,17], Phrap (http://www.phrap.org/) and Consed [18]. Sequences of the 83 isolates were identified at the genus level based on blastn of the National Center for Biotechnology Information (NCBI) and deposited in the GenBank database. The accession numbers of 16S rRNA sequences are available in Table S2. After molecular characterization of each isolate, the diversity of genera in each sampling site was analysed by Shannon’s index (H’) [19], using the software Statistica [20].

### Phylogenetic reconstruction of the 16S rRNA gene of plant growth-promoting bacteria

Multiple sequence alignments of the 83 isolates, as well as the type strains of the closest species, were obtained with muscle [21]. To select the closest type strains to our sequences, we used the blastn alignments with the highest identity, and the closest type strains were detected in the phylogenies assessed with the type strain genome server [22] (data not shown). The best evolutionary distance model was defined with the lowest Bayesian information criterion scores for maximum-likelihood reconstructions on the Molecular Evolutionary Genetics Analysis (mega) software version 7 [23]. The statistical support bootstrap was evaluated using 1,000 replicates [24]. Nucleotide identity (NI) matrices were calculated with BioEdit version 7.0.4.1 [25].

### Production of indolic compounds

Each 1 of the 83 isolates was inoculated in DYGS medium broth [14] with (in triplicate) or without (only once) 100 µg ml^−1^ of tryptophan and incubated for 24 h at 30 °C in the dark, at 120 r.p.m. Bacterial growth was evaluated as OD in a spectrophotometer at 600 nm. Subsequently, 2 ml of each bacterial growth was transferred to microtubes and centrifuged at 10,000 ***g*** for 10 min, and then 1.5 ml of the supernatant was transferred to vials and received 1 ml of Salkowski’s reagent [26]. After 30 min in the dark at room temperature, the colour intensity was measured at 540 nm, and the results were expressed as µg ml^−1^ of indoleacetic acid (IAA) based on a calibration curve using synthetic IAA.

### Putative biological nitrogen fixation capacity *in vitro*

After five consecutive stabbing steps in the respective N-free semi-solid culture medium, the isolates were assessed for putative biological nitrogen fixation (BNF) capacity *in vitro* based on the N enrichment in the corresponding culture medium [12] from which they were isolated, after 7 days of growth at 30 °C [27]. At all inoculation steps, the isolates were checked for the formation of a typical pellicle beneath the surface of the respective semi-solid culture medium [12]. After incubation, the culture media were homogenized, and 3 ml was digested in sulfuric acid, following the semi-micro-Kjeldahl distillation [28] for the determination of N enrichment in the culture media. This assay was evaluated without replicates.

### P-solubilization and production of siderophores

Each isolate was grown on a culture medium containing tricalcium phosphate for checking the P-solubilization ability [29] after 3 days of incubation at 30 °C in the dark, in triplicate. The presence of a clear halo around the colony was considered P-solubilizing positive, which was measured with a pachymeter on the sixth day of incubation. For production of siderophores, the isolates were grown on solidified DYGS medium [14] containing chromeazurol S (chromeazurol S 60.5 mg l^−1^, hexadecyltrimethylammonium 72.9 mg l^−1^ and 1 mM iron chloride in 10 mM HCl) [30], in triplicate. The presence of a halo was considered a siderophore producer and was measured with a pachymeter after 3 days of incubation.

### Effects of the bacterial isolates on the growth promotion of maize plantlets

Based on taxonomic identification and plant growth-promoting traits *in vitro*, at least one isolate of each genus with the most promising results was selected to be tested for growth promotion capacity in maize plantlets, amounting to 23 isolates. Seeds of the landrace cv. Caiano were surface-disinfected (70% ethanol, 3 min; 20% commercial hypochlorite, 5 min; rinsing with sterile water) and germinated under aseptic conditions in a germination chamber. Three-day-old maize plantlets were transferred to 200 ml of sterile hydroponic Hoagland’s nutrient solution [31] without mineral N. Subsequently, 1 ml of each bacterial suspension at O.D._600_=0.5 (~10^8^ cells ml^−1^) was inoculated on the plantlets’ roots, in six replications per isolate. Negative control without inoculation and a positive control with the commercial strain Ab-V5 of *Azospirillum brasilense* were included. After 10 days of growth in the greenhouse, plantlets were evaluated for shoot and root dry biomasses (g), root length (m) and root-specific length (m g^−1^). Data were submitted to ANOVA and Scott–Knott test (*P*≤0.10) after transformation to the root square and presented as relative difference (%) from the average non-inoculated control.

### Genomic DNA extraction and genome sequencing of promising strains

The genomic DNA of the two most promising strains CNPSo 2378 and CNPSo 2384 was extracted using the DNeasy Blood and Tissue kit (QIAGEN) following the manufacturer’s instructions. Libraries were constructed with the Nextera XT kit according to the manufacturer’s protocol and sequenced on the NextSeq 1000 (Illumina) at Embrapa Soja, Londrina-PR, Brazil. The reads were assembled with the A5-MiSeq pipeline (*de novo*) version 20140604 [32], and the draft genomes were deposited at the NCBI (accession number JAXCMJ000000000 for CNPSo 2378; JAXCMK000000000 for CNPSo 2384). Genome assemblies were annotated using the NCBI Prokaryotic Genome Annotation Pipeline program [33]. The plant growth-promoting genes were detected according to the genomes of *Bacillus velezensis* FZB42 [4] and *Sphingomonas* LK11 [34]. The average nucleotide identity (ANI) [35] and digital DNA–DNA hybridization (dDDH) [36] were calculated with the closest type strains.

## Results

### 16S rRNA sequence analyses and frequency of genera

A total of 83 endophytic strains were isolated, being 36 in JNFb, 26 in JMV and 21 in LGI culture medium. The isolates were deposited in the Diazotrophic and Plant Growth-Promoting Bacteria Culture Collection (WFCC Collection No. 1213, WDCM Collection No. 1054) at Embrapa Soja (CNPSo), Londrina, PR, Brazil [37]. The respective identification in the culture collection (CNPSo code) and the accession number of the 16S rRNA sequences deposited at GenBank are provided in Table S2. The identified strains belonged to 14 genera of the classes *α-Proteobacteria*, *β-Proteobacteria*, *γ-Proteobacteria*, *Actinomycetia* and *Bacilli* (Table S2). From six to eight genera were isolated in each sampling site (Tables 1 and S2). The genera *Agrobacterium, Burkholderia* and *Stenotrophomonas* were isolated in all the three agricultural systems, characterized by a transition from conventional to organic system (sites 1 and 2), organic system (site 3) and low-input conventional systems (sites 4 and 5), whereas some genera were isolated in only one site: *Bacillus* (site 5), *Microbacterium* (site 1), *Pseudomonas* (site 1) and *Xanthomonas* (site 3) (Table S2).

**Table 1.** Taxonomic composition of the 83 endophytic bacteria isolated from maize grown in different agricultural systems

	Agricultural system			
Class/genera	Transitional (sites 1 and 2)	Organic (site 3)	Low-input conventional (sites 4 and 5)	Total in five sites
	**Number of strains**			
***α-Proteobacteria***				
*Agrobacterium*	6	2	4	12
*Rhizobium*	3	–	3	6
*Sphingomonas*	1	1	1	3
***β-Proteobacteria***				
*Burkholderia*	9	7	5	21
*Paraburkholderia*	2	–	3	5
***γ-Proteobacteria***				
*Pseudomonas*	1	–	–	1
*Stenotrophomonas*	4	1	3	8
*Phytobacter*	2	3	–	5
*Enterobacter*	1	–	6	7
*Klebsiella*	–	1	1	2
*Pseudoxanthomonas*	–	3	3	6
*Xanthomonas*	–	1	–	1
***Actinomycetia***				
*Microbacterium*	4	–	–	4
***Bacilli***				
*Bacillus*	–	–	2	2
**Total**	**33**	**19**	**31**	**83**

The most abundant genus was *Burkholderia*, with 21 strains*,* followed by *Agrobacterium* with 12 strains and *Stenotrophomonas* with 8 strains. On the other hand, the least abundant genera were *Bacillus* and *Klebsiella*, with two strains each, and *Pseudomonas* and *Xanthomonas*, both with one strain each (Table S2). Shannon’s index of diversity did not differ among the sampling sites (not shown). However, the highest number of strains was detected in the organic cropping system (site 3) with 19 strains belonging to 8 different genera, followed by site 2 (transitional), with 18 strains belonging to 6 different genera; site 5 (low-input conventional), with 16 strains of nine genera; and sites 1 and 4, each with 15 strains belonging to 7 and 6 different genera, respectively (Tables 1 and S2).

The phylogenetic trees were constructed according to the identified classes; therefore, five trees were constructed. Fig. S1 represents the *α-Proteobacteria* class tree containing 12 *Agrobacterium* strains, 6 *Rhizobium* strains and 3 *Sphingomonas* strains, in addition to the type strains of the closest species. Within *Agrobacterium*, *Rhizobium* and *Sphingomonas*, the strains shared 98–100%, 99.7–99.9% and 98.2–99.2% similarity, respectively, with 16S rRNA sequences deposited at GenBank. In addition, it was possible to observe different evolutionary histories of some strains in the phylogenetic tree. Concerning the *Agrobacterium* genus, eight strains were closely related to *Agrobacterium pusense* NRCPB10^T^ and *Agrobacterium salinitolerans* YIC 5082^T^, sharing 99.8 and 99.9% similarity, respectively. The strain CNPSo 2368 grouped with 99% of bootstrap support with ‘*Agrobacterium fabrum*’ C58^T^ sharing 99.8% similarity; and the strains CNPSo 2312 and CNPSo 2367 were also closely related to ‘*Agrobacterium fabrum*’ C58^T^ type strain, with 99.1 and 99.2% similarity, respectively. The strain CNPSo 2346 was closely related to *Agrobacterium arsenijevicii* KFB 330^T^, *Agrobacterium fabacearum* CNPSo 675^T^ and *Agrobacterium tumefaciens* ATCC 4720^T^, with 99% bootstrap support and 100% similarity. The six strains of *Rhizobium* were positioned in the *Rhizobium tropici* clade, with 86% bootstrap support and NI values ranging from 99.2 to 99.7% similarity with *Rhizobium freirei* PRF 81^T^ and *Rhizobium tropici* CIAT 899^T^. One of the *Sphingomonas* strains, CNPSo 2363, grouped with *Sphingomonas paucimobilis* DSM 30198^T^, sharing 98.9% similarity, whereas CNPSo 2323 and CNPSo 2378 were closely related to *Sphingomonas sanguinis* NBRC 13937^T^ and *Sphingomonas parapaucimobilis* NBRC 15100^T^ and shared 99.6 and 99% similarity with each type strain, respectively.

The *β-Proteobacteria* class encompassed 21 *Burkholderia* strains and five *Paraburkholderia* strains (Fig. S2). NI values among the strains within the *Burkholderia* and *Paraburkholderia* genera ranged from 97.1 to 100% and 95.6 to 99.6% similarity, respectively. Basically, the *Burkholderia* strains were grouped into two different clusters. The clade I included 15 strains and *Burkholderia gladioli* CIP 105410^T^, *Burkholderia perseverans* INN12^T^ and *Burkholderia plantarii* CIP 105769^T^ that shared NI values ranging from 98.1 to 99.3% similarity. The clade II grouped six strains and *Burkholderia cenocepacia* LMG 16656^T^, *Burkholderia reimsis* BE51^T^, *Burkholderia contaminans* J2956^T^ and *Burkholderia cepacia* ATCC 25416^T^, sharing from 99.1 to 99.8% similarity with the type strains. Regarding *Paraburkholderia*, two strains showed higher relatedness with *Paraburkholderia tropica* Ppe8^T^ and three others with *Paraburkholderia caribensis* MWAP64^T^, sharing 99.9 and 99.6% similarity.

Fig. S3 shows the *γ-Proteobacteria* class, with eight *Stenotrophomonas* strains, seven *Enterobacter*, six *Pseudoxanthomonas*, five *Phytobacter*, two *Klebsiella*, one *Pseudomonas* strain and one *Xanthomonas*. In general, the phylogenetic tree was divided into three large clades (Fig. S3). The clade I grouped strains belonging to the genera *Enterobacter*, *Phytobacter* and *Klebsiella*, with 100% bootstrap support; the strains within each genus shared NI values ranging from 98.7to 99.9%, 99.4 to 100% and 97.7% similarity among each other, respectively. Concerning the *Enterobacter* genus, the strains were closely related to type strains of several species, such as *Enterobacter huaxiensis* 090008^T^, *Enterobacter mori* LMG 25706^T^, *Enterobacter roggenkampii* DSM 16690^T^, *Enterobacter wuhouensis* WCHEs120002^T^, *Enterobacter vonholyi* E13^T^ and *Enterobacter quasiroggenkampii* WCHECL1060^T^. The five *Phytobacter* strains presented *Phytobacter diazotrophicus* DSM 17806^T^ as the closest type strain. *Klebsiella* CNPSo 2359 grouped with *Klebsiella variicola* DSM 15968^T^, whereas *Klebsiella* CNPSo 2392 grouped with *Klebsiella grimontii* SB73^T^ and *Klebsiella michiganensis* W14^T^. Clade II, with 99% bootstrap support, included the *Pseudomonas* CNPSo 2319 and close type strains of the genus, being *Pseudomonas chengduensis* MBR the closest type strain with 98.6% similarity. The clade III grouped the genera *Pseudoxanthomonas, Stenotrophomonas* and *Xanthomonas* with 100% bootstrap support. The six *Pseudoxanthomonas* presented the highest relatedness with *Pseudoxanthomonas winnipegensis* NML 130738, with NI values of 99.8 and 100% similarity. Concerning the *Stenotrophomonas* strains, they showed variability in the 16S rRNA sequences among each other; four strains were closer to *Stenotrophomonas maltophilia* DSM 50170^T^, and the other four strains were closer to *Stenotrophomonas geniculata* ATCC 19374^T^, with NI values ranging from 98.9 to 99.3% and 98.7 to 99.8% similarity. Finally, the *Xanthomonas* strain CNPSo 2366 was closely related to *Xanthomonas albilineans* LMG 494^T^ (99.1%) and *Xanthomonas sacchari* DSM 22617^T^ (99.5%) and shared NI values of 99.1% and 99.5% similarity with those type strains, respectively.

The *Actinomycetia* class included only four *Microbacterium* strains closely related to each other, with NI values ranging from 99.6 to 99.9% of similarity, which were grouped with 100% bootstrap support with *Microbacterium binotii* CIP 101303^T^ and *Microbacterium neimengense* 7087^T^ and shared, respectively, from 99.7 to 100% and from 99.6 to 99.8% similarity (Fig. S4). The two strains identified as *Bacillus,* belonging to the *Bacilli* class, are presented in Fig. S5. *Bacillus* CNPSo 2383 was closely related to *Bacillus velezensis* subsp. *plantarum* FZB42 (=*Bacillus amyloliquefaciens*), whereas *Bacillus* CNPSo 2384 was related to *Bacillus velezensis* CBMB205^T^ (=*Bacillus methylotrophicus*), *Bacillus siamensis* KCTC 13613^T^ and *Bacillus velezensis* NRRL B-41580^T^. The strains CNPSo 2383 and CNPSo 2384 shared 99.6% similarity and 99.8% and 99.6% with *Bacillus velezensis* subsp. *plantarum* FZB42 (=*Bacillus amyloliquefaciens*), respectively, 99.7% with *Bacillus velezensis* CBMB205^T^ (=*Bacillus methylotrophicus*), 99.8% *Bacillus siamensis* KCTC 13613^T^ and 99.7% with *Bacillus velezensis* NRRL B-41580^T^.

### Production of indolic compounds, putative BNF, P-solubilization and siderophores

The synthesis of indolic compounds was observed ubiquitously among the strains under investigation, especially in the presence of tryptophan compared with no tryptophan added to the growth media (Table S3). Notably, strains CNPSo 2374, CNPSo 2376, CNPSo 2343 and CNPSo 2391 of *Enterobacter*, *Klebsiella* CNPSo 2359, *Stenotrophomonas* CNPSo 2326 and *Burkholderia* CNPSo 2334 were outstanding in the synthesis of indolic compounds in the presence of tryptophan, most of them producing equivalent or above the positive control with *Azospirillum brasilense* Ab-V5, which yielded 16.04±2.72 µg ml^−1^. Noteworthy was the synthesis of indolic compounds without added tryptophan by *Stenotrophomonas* CNPSo 2326, reaching 6.44 µg ml^−1^, and *Bacillus* CNPSo 2384, producing 4.13 µg ml^−1^. Other strains were also capable of synthesizing indolic compounds without added tryptophan compared with the commercial strain of *Azospirillum brasilense* Ab-V5 (Table S3).

Regarding putative BNF capacity, all strains showed the ability to enhance N concentration in N-free culture media at different levels (Table S3). Strains of *Burkholderia* CNPSo 2340, *Rhizobium* CNPSo 2320, *Paraburkholderia* CNPSo 2385, *Enterobacter* CNPSo 2375, *Klebsiella* CNPSo 2359 and *Pseudoxanthomonas* CNPSo 2381 exhibited outstanding increases in N concentration, reaching up to 30.8, 25.2, 23.8, 19.6, 16.8 and 16.8 µg ml^−1^, respectively.

The genera *Burkholderia* (19 out of 21 strains) and *Paraburkholderia* (2 out of 5 strains) were the only capable of P-solubilization, with haloes extending up to 11.2±0.2 mm and 9±0.2 mm, respectively (Table S3).

Except for the genus *Microbacterium*, which did not produce siderophores, siderophore production was a widespread trait among the strains investigated, with halos reaching 48.4 mm in *Burkholderia* CNPSo 2313, 42.3 mm in *Agrobacterium* CNPSo 2330, 41.5 mm in *Xanthomonas* CNPSo 2366 and 39.7 mm in *Paraburkholderia* CNPSo 2385 (Table S3).

### Growth promotion of maize plantlets

**Fig. 1.**
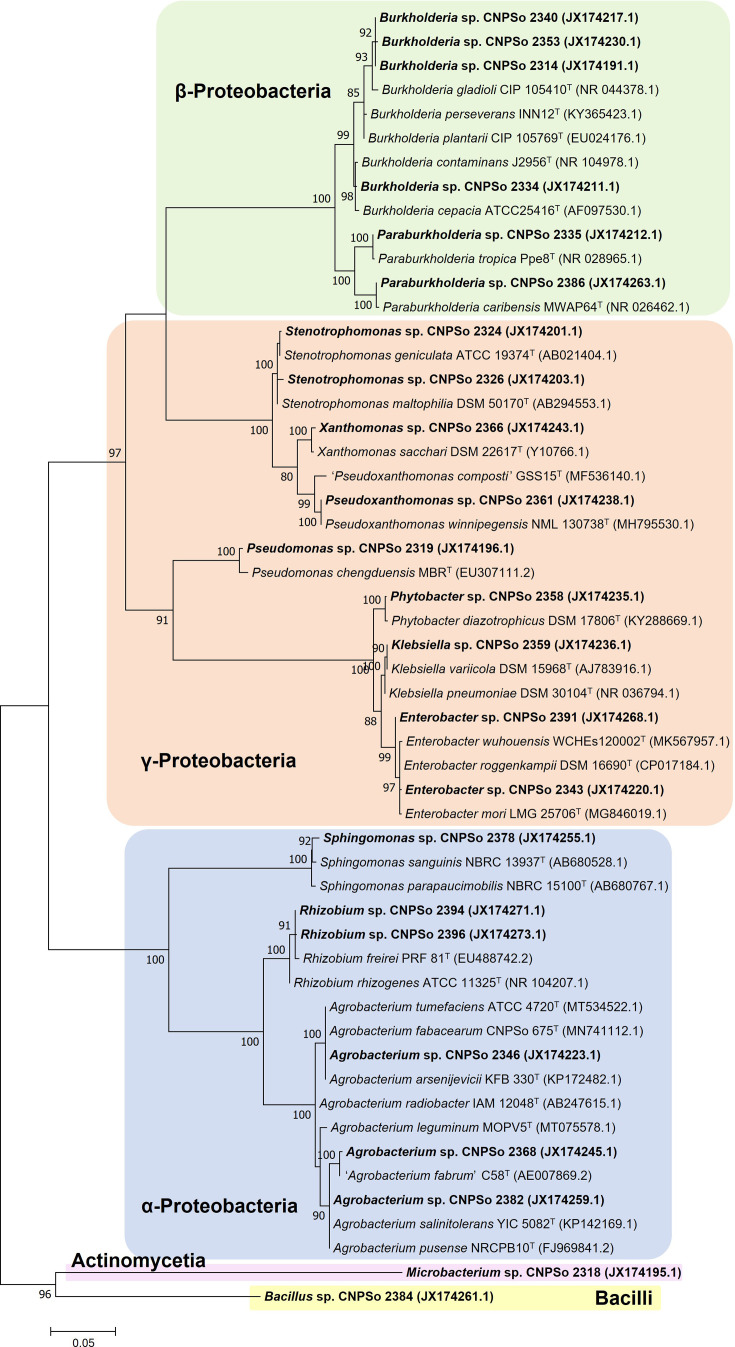
Maximum-likelihood phylogeny based on the 16S rRNA gene alignment (1,218 bp), using the Tamura–Nei model+G by mega v. 7. Accession numbers are indicated in parentheses and in Table S1. The promising strains are shown in bold. Bootstrap values >70% are indicated at the nodes. Bar indicates the percentage of nucleotide substitutions.

A total of 23 strains were selected based on the taxonomic identification and on *in vitro* plant growth-promoting traits (Table S3) to assess their efficiency in promoting the growth of maize plantlets. Among these promising strains, four were identified as *Burkholderia* (CNPSo 2314, 2334, 2340 and 2353), three as *Agrobacterium* (CNPSo 2346, 2368 and 2382), two of each as *Rhizobium* (CNPSo 2394 and 2396), *Paraburkholderia* (CNPSo 2335 and 2386), *Stenotrophomonas* (CNPSo 2324 and 2326) and *Enterobacter* (CNPSo 2343 and 2391) and one of each as *Phytobacter* (CNPSo 2358), *Sphingomonas* (CNPSo 2378), *Pseudomonas* (CNPSo 2319), *Klebsiella* (CNPSo 2359), *Pseudoxanthomonas* (CNPSo 2361), *Xanthomonas* (CNPSo 2366), *Microbacterium* (CNPSo 2318) and *Bacillus* (CNPSo 2384) (Fig. 1).

**Fig. 2.**
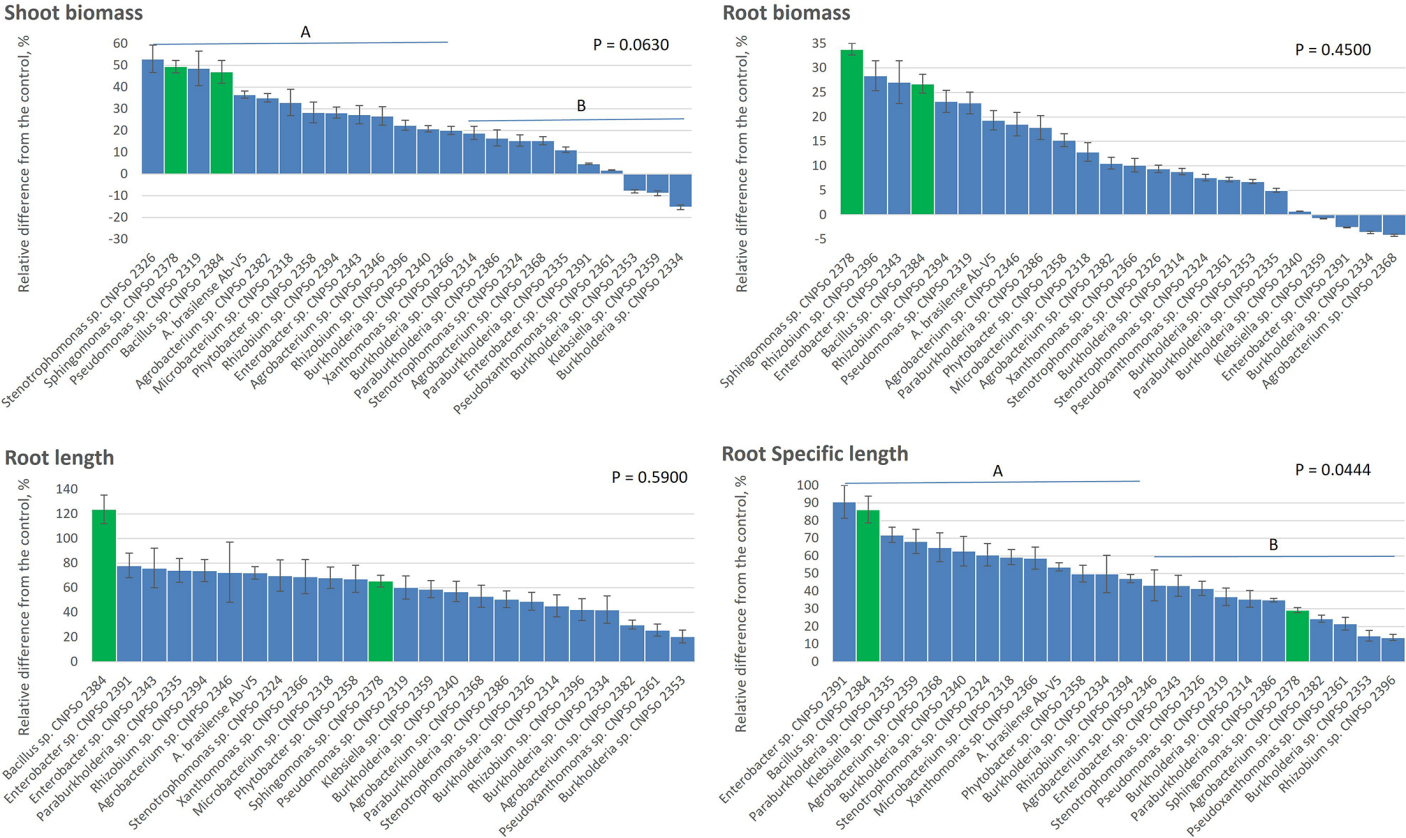
Relative difference (%) for the initial growth (shoot biomass, root biomass, root length and root-specific length) of maize inoculated with promising isolates of endophytic bacteria isolated from maize cropped under organic and low-input agricultural systems in southern Brazil and a reference commercial strain of *Azospirillum brasilense* (Ab-V5) (*n*=6). Horizontal bars with different letters represent distinct groups by the Scott–Knott test (*P*≤0.10).

**Fig. 3.**
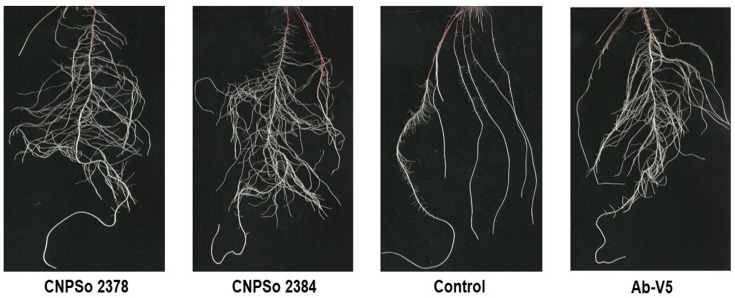
Response of maize roots ten days after inoculation with two promising isolates of endophytic bacteria, *Sphingomonas* CNPSo 2378 or *Bacillus velezensis* CNPSo 2384. Non-inoculated and positive control with *Azospirillum brasilense* Ab-V5 are included.

The majority of isolates enhanced the maize plantlets’ growth, reaching by nearly 50% shoot biomass and around 30% root biomass over the non-inoculated control when inoculated with *Sphingomonas* CNPSo 2378 or *Bacillus* CNPSo 2384 (Fig. 2). Significant effects were observed for shoot biomass and root-specific length, with two groupings of strains (Fig. 2). Some isolates promoted negative effects on shoot biomass like *Burkholderia* CNPSo 2334 and CNPSo 2353 and *Klebsiella* CNPSo 2359, whereas others were negative only for root biomass, like *Agrobacterium* CNPSo 2368 and *Enterobacter* CNPSo 2391. Despite this, all isolates improved the root length (up to 120% with *Bacillus* CNPSo 2384 and over 60% with *Sphingomonas* CNPSo 2378) and root-specific length (up to 90% with *Enterobacter* CNPSo 2391, followed by *Bacillus* CNPSo 2384) (Fig. 2). Therefore, the strains *Sphingomonas* CNPSo 2378 and *Bacillus* CNPSo 2384 showed promising results in terms of root morphological (Fig. 3) and plant growth-promoting traits (Table S3).

### Genome statistics of promising strains CNPSo 2378 and CNPSo 2384

The two promising plant growth-promoting strains, *Sphingomonas* CNPSo 2378 and *Bacillus* CNPSo 2384, were selected for genome sequencing. More information on the genome assemblies is provided in Table S4.

The draft genome of *Sphingomonas* CNPSo 2378 was estimated at 4,331,468 bp with 184 contigs and an N50 of 77,510. The genome coverage and guanine and cytosine content (G+C%) were estimated to be 381-fold and 66.43%, respectively. The genome annotation presented 3,843 coding sequences, 66 pseudogenes and 61 RNA sequences. Comparing the genomes of the strain CNPSo 2378 with its closely related strains *Sphingomonas sanguinis* NBRC 13937 and *Sphingomonas paraucimobilis* NBRC 15100 (Figs 1 and S1), this promising strain shared 86.1% and 77.39% of ANI with the respective type strains and 31.1% and 31.2% of dDDH, respectively (Table S4).

The genome of *Bacillus velezensis* CNPSo 2384 was estimated at 4,133,224 bp with 74 contigs and an N50 of 194,138. The genome coverage and guanine and cytosine content (G+C%) were estimated to be 415-fold and 46.02%, respectively. The annotation revealed 4,051 coding sequences, 89 pseudogenes and 95 RNA sequences. According to ANI and dDDH genomic comparisons (Table S4), the strain CNPSo 2384 was classified as *Bacillus velezensis*, sharing 98.93% and 90.6% with *Bacillus velezensis* subsp. *plantarum* FZB42 (=*Bacillus amyloliquefasciens*), 98.37% and 85% with *Bacillus velezensis* NRRL-B41580 and 98.26% and 84.4% with *Bacillus velezensis* KACC 13105 (=*Bacillus methylotrophicus*), the closest type strains in the 16S rRNA phylogenies (Fig. S5).

The annotation of both genomic sequences revealed the presence of several genes potentially involved in plant growth-promoting traits. More specifically, *Sphingomonas* CNPSo 2378 genome presented genes related to tryptophan biosynthesis, P-solubilization and siderophore uptake, whereas CNPSo 2384 has genes involved with IAA biosynthesis, P-solubilization, siderophore uptake and biocontrol. Both genomes showed the presence of a unique gene involved in the N-fixing process, encoding the NifU protein. Information about these genes of both promising strains was presented in Table S5.

## Discussion

Agricultural practices impact microbial composition within agroecosystems, and some studies have consistently highlighted that organically managed systems exhibit enhanced microbial communities [10,11, 38]. Within the soil, a diverse array of bacteria holds several mechanisms that may help plants to reach their nutritional requirements and control phytopathogens. These bacteria, known as plant growth promoters, may exist as free-living, as rhizosphere or root surface associated, as endophytes within plant tissues or as symbionts of plants [1,3, 7]. In this study, we isolated 83 endophytic strains with plant growth-promoting traits from landrace maize varieties grown in five distinct sites characterized by low-input conventional, transitional from conventional to organic, and organic management systems.

Even though we have not detected significant differences in the bacterial diversity from maize grown under different agricultural practices, using culture-based methods, the isolates were classified into 14 genera across *α-Proteobacteria*, *β-Proteobacteria, γ-Proteobacteria*, *Actinomycetia* and *Bacilli* classes (Fig. 1, Table 1). Each site harboured from six to eight different genera, with certain genera being ubiquitous across all sites, while others were site-specific. This suggests that agricultural practices affect the composition and distribution of bacterial communities endophytically associated with maize plants. The wide diversity of bacterial genera detected shows members ubiquitous in the soil microbial community, and several of them are recognized as plant growth promoters. Most isolated exhibited key plant growth-promoting traits, such as nutrient acquisition, siderophores and phytohormone biosynthesis *in vitro* (Table S3), as well as promoted the growth of maize plantlets (Fig. 2), underscoring their role in enhancing plant performance in the production system.

Despite the outstanding plant growth promotion effect evidenced by several strains, some of them were classified as potentially pathogenic for humans, animals or plants. *Burkholderia* encompasses clinically important species within the *β-Proteobacteria* class [39]**,** presenting strains with notable enhancement of N concentration in N-free culture media, P-solubilization and siderophore production (Table S3). However, these strains, such as CNPSo 2313 and CNPSo 2340, were phylogenetically clustered, among several other isolates, within the *Burkholderia cepacia* complex (Fig. S2), which includes opportunistic pathogenic bacteria involved in respiratory infections [40]. Other examples are *Enterobacter* CNPSo 2391 and *Stenotrophomonas* CNPSo 2326, with noteworthy results for IAA biosynthesis with and without tryptophan *in vitro*, respectively, but these genera are frequently associated with opportunistic and multidrug-resistant pathogens for humans [41,42]. *Agrobacterium* and *Xanthomonas* presented one strain each with outstanding production of siderophore *in vitro* (Table S3); however, both genera include phytopathogens related to considerable economic losses in several crops [43]. For this reason, further investigations are required to assess their pathogenic potential before considering their use as bioinput.

Regarding the growth promotion of maize plantlets, the strains *Sphingomonas* CNPSo 2378 and *Bacillus* CNPSo 2384 exhibited remarkable increases in shoot (49 and 47%, respectively) and root biomasses (34 and 27%), root length (65 and 124%) and root-specific length (29 and 86%), over the non-inoculated control, with values similar to the commercial strain Ab-V5 of *Azospirillum brasilense*, except *Sphingomonas* CNPSo 2378 for root-specific length (Figs2  3). Due to this evidence on the capacity of promoting maize plantlets’ growth, the genomes of both strains were sequenced to obtain an accurate taxonomic identification and search for genes potentially involved in plant growth-promoting traits (Table S4).

Concerning the taxonomic analysis based on genomic sequences, the strain *Sphingomonas* CNPSo 2378 exhibited ANI and dDDH values below the threshold for delimitation of species [44] when compared with the closely related *Sphingomonas sanguinis* NBRC 13937 and *Sphingomonas paraucimobilis* NBRC 15100, suggesting that this strain might represent a novel species within the *Sphingomonas* genus (Table S4). However, the strain *Bacillus velezensis* CNPSo 2384 was identified as belonging to the species *Bacillus velezensis* for presenting ANI and dDDH values greater than 98.26% and 84.4%, respectively (Table S4).

Bacterial strains can stimulate plant growth by biosynthesizing of indolic compounds that stimulate cell division, elongation and formation of root hair. The synthesis of indolic compounds in bacteria may occur through pathways relying on tryptophan, which is the main precursor of IAA, or operate independently [45]. The strain *Sphingomonas* CNPSo 2378 synthesized indolic compounds either with or without tryptophan added to the culture medium; however, only genes involved in the biosynthesis of tryptophan (*trpABCDE*) were found (Table S5). Genes involved in the synthesis of tryptophan were previously reported in the genome of *Sphingomonas* LK11, a putative plant growth-promoting strain [34]. The absence of genes related to IAA biosynthesis may be potentially attributed to the lack of studies focused on those genes of *Sphingomonas* strains so far. Concerning *Bacillus velezensis* CNPSo 2384, functional analysis of the genome revealed the genes: *patB*, encoding an aminotransferase; *yhcX*, a nitrilase; *dhaS*, indole-3-acetaldehyde dehydrogenase; *ysnE*, tryptophan acetyltransferase; *yclB*, an aromatic-acid decarboxylase; and *yclC*, located in the same operon as *yclB*, encoding a UbiD family decarboxylase (Table S5). It has been suggested that these six genes are involved in the pathways of indole-3-pyruvic acid, indole-3-acetamide, indole-3-acetonitrile and tryptamine, as well as an uncharacterized IAA biosynthesis pathway, and significantly influenced the biosynthesis of IAA by *Bacillus velezensis* strain SQR9 [46]. Additionally, the presence of *trpABCDE* genes in *Bacillus velezensis* CNPSo 2384, which is involved in the synthesis of tryptophan, corroborates the second-highest ability of this strain among the isolates in producing IAA without the addition of tryptophan in the culture medium (Tables S3 and S5).

Although in our evaluation the promising strains *Sphingomonas* CNPSo 2378 and *Bacillus velezensis* CNPSo 2384 were not able to solubilize inorganic phosphate (tricalcium phosphate) *in vitro*, several putative genes related to P-solubilization were reported in their genomes. *Sphingomonas* CNPSo 2378 presented genes within the PHO operon (*phoURB*) and phosphate-specific transporters (*pstABCS*) [47], whereas a phosphate ABC transporter system (*pstABC*), a PhoH family protein and *phyC* gene, encoding an isoform of the enzyme phytase, responsible for catalysing the hydrolysis of phytic acid, releasing phosphate and inositol [1] were detected in the genome of *Bacillus velezensis* CNPSo 2384 (Table S5). The inability to solubilize phosphate despite the presence of these genes may indicate either a lack of gene expression *in vitro* or that the product of these genes may act on other phosphates like Fe- and Al-phosphates [48], which were not tested in this study.

A total of 102 genes encoding TonB-dependent receptors or part of them were detected in the genome of *Sphingomonas* CNPSo 2378, which are outer-membrane proteins responsible for the uptake of siderophores and other substrates from the environment [49]. In addition to supplying iron nutrition for plants, siderophore-producing bacteria may reduce the competitiveness of plant pathogens by immobilizing iron from the surrounding environment [1]. Still about suppressing plant pathogens, the closest strain to *Bacillus velezensis* CNPSo 2384, *Bacillus velezensis* FZB42, is known for its capacity for biocontrol, which is attributed to nine giant gene clusters responsible for synthesizing a diverse array of bioactive secondary metabolites, facilitated by non-ribosomal peptide synthetases and polyketide synthases. These bioactive compounds play pivotal roles in biocontrol, effectively targeting fungi, bacteria and other microbial competitors [4]. We detected eight out of these nine gene clusters in the genome of *Bacillus velezensis* CNPSo 2384 (Table S5). The gene clusters *srf*, *bmy*, *fen* and *dhb* are involved in the synthesis of cyclic lipopeptides, such as surfactin, bacillomycin-D, fengycin and the iron-siderophore bacillibactin, respectively, while the four other clusters *bac*, *mln*, *bae* and *dfn* were reported to drive the synthesis of antibacterial dipeptides and polyketides, such as bacilysin, macrolactin, bacillaene and difficidin, respectively [4,5]. The *nrs*, coding an unknown peptide, was the only cluster that we did not find. These findings indicate that *Bacillus velezensis* CNPSo 2384 might show biocontrol activity, being an interesting strain with multifunctional purposes of agricultural interest.

The unique gene related to BNF on both *Sphingomonas* CNPSo 2378 and *Bacillus velezensis* CNPSo 2384 genomes was *nifU* (Table S5), whose protein has been studied mainly in *Azotobacter vinelandii*, being required for full activation of the nitrogenase and also as a provider of [Fe-S] clusters that serve as metabolic substrates for the synthesis of the cofactor FeMo [50]. Therefore, *nifU* might play a role in the activation of other enzymes than in the nitrogenase complex. The gene *nifU* was also present in the genome of *Bacillus velezensis* FZB42, the closest strain of *Bacillus velezensis* CNPSo 2384.

## Conclusions

We isolated and identified a diverse array of bacterial strains belonging to 14 genera living endophytically in maize grown across 3 different agricultural management sites, ranging from low-input conventional to organic. These results suggest that low-input and organic cropping systems are hotspots for isolation of bacterial strains with biotechnological potential, presenting a range of functional traits for plant growth promotion. Inoculation with selected strains significantly enhanced the initial growth of maize plantlets. Particularly promising results were observed with the strains *Sphingomonas* sp. CNPSo 2378 and *Bacillus velezensis* CNPSo 2384, whose genome sequencing provided valuable insights into their genetic composition and relatedness to known species.

Further research exploring the mechanisms underlying plant–microbe interactions and the feasibility of using these bacterial strains as agricultural bio-inputs are in course for their effective implementation.

## Supplementary material

10.1099/acmi.0.000948.v3 Supplementary Material 1.

## Abbreviations

ANI: average nucleotide identity
BNF: biological nitrogen fixation
dDDH: digital DNA–DNA hybridization
IAA: indoleacetic acid
mega: Molecular Evolutionary Genetics Analysis
NCBI: National Center for Biotechnology Information
NI: nucleotide identity
PGPB: plant growth-promoting bacteria

## References

[1] Glick BR (2020). Beneficial Plant-Bacterial Interactions, 2nd ed.

[2] Hungria M, Nogueira MA, Rengel Z, Cakmak I, White PJ (2022). Marschner’s Mineral Nutrition of Plants.

[3] Fukami J, Cerezini P, Hungria M (2018). *Azospirillum*: benefits that go far beyond biological nitrogen fixation. AMB Express.

[4] Chen XH, Koumoutsi A, Scholz R, Eisenreich A, Schneider K (2007). Comparative analysis of the complete genome sequence of the plant growth-promoting bacterium *Bacillus amyloliquefaciens* FZB42. Nat Biotechnol.

[5] Rabbee MF, Ali MS, Choi J, Hwang BS, Jeong SC (2019). *Bacillus velezensis*: a valuable member of bioactive molecules within plant microbiomes. Molecules.

[6] Klepa MS, diCenzo GC, Hungria M (2024). Comparative genomic analysis of *Bradyrhizobium* strains with natural variability in the efficiency of nitrogen fixation, competitiveness, and adaptation to stressful edaphoclimatic conditions. Microbiol Spectr.

[7] Hungria M, Barbosa JZ, Rondina ABL, Nogueira MA (2022). Improving maize sustainability with partial replacement of N fertilizers by inoculation with *Azospirillum brasilense*. Agronomy J.

[8] Singh R, Goodwin SB (2022). Exploring the corn microbiome: a detailed review on current knowledge, techniques, and future directions. PhytoFront.

[9] Youseif SH (2018). Genetic diversity of plant growth promoting rhizobacteria and their effects on the growth of maize plants under greenhouse conditions. Ann Agric Sci.

[10] Shu W, Pablo GP, Jun Y, Danfeng H (2012). Abundance and diversity of nitrogen-fixing bacteria in rhizosphere and bulk paddy soil under different duration of organic management. World J Microbiol Biotechnol.

[11] Xia Y, DeBolt S, Dreyer J, Scott D, Williams MA (2015). Characterization of culturable bacterial endophytes and their capacity to promote plant growth from plants grown using organic or conventional practices. Front Plant Sci.

[12] Baldani JI, Reis VM, Videira SS, Boddey LH, Baldani VLD (2014). The art of isolating nitrogen-fixing bacteria from non-leguminous plants using N-free semi-solid media: a practical guide for microbiologists. Plant Soil.

[13] Ormeño-Orrillo E, Hungria M, Martínez-Romero E, Rosemberg E, Long EF, Lory S, Stackebrandt E, Thompson F (2013). The Prokaryotes - Prokaryotic Physiology and Biochemistry.

[14] Fukami J, Abrantes JLF, del Cerro P, Nogueira MA, Ollero FJ (2018). Revealing strategies of quorum sensing in *Azospirillum brasilense* strains Ab-V5 and Ab-V6. Arch Microbiol.

[15] Menna P, Hungria M, Barcellos FG, Bangel EV, Hess PN (2006). Molecular phylogeny based on the 16S rRNA gene of elite rhizobial strains used in Brazilian commercial inoculants. Syst Appl Microbiol.

[16] Ewing B, Green P (1998). Base-calling of automated sequencer traces using phred. II. Error probabilities. Genome Res.

[17] Ewing B, Hillier L, Wendl MC, Green P (1998). Base-calling of automated sequencer traces using phred. I. Accuracy assessment. Genome Res.

[18] Gordon D, Abajian C, Green P (1998). Consed: a graphical tool for sequence finishing. Genome Res.

[19] Magurran AE (1988). Ecological Diversity and Its Measurement.

[20] StatSoft Inc (2004).

[21] Edgar RC (2004). MUSCLE: multiple sequence alignment with high accuracy and high throughput. Nucleic Acids Res.

[22] Meier-Kolthoff JP, Göker M (2013). TYGS is an automated high-throughput platform for state-of-the-art genome-based taxonomy. Nat Commun.

[23] Kumar S, Stecher G, Tamura K (2016). MEGA7: Molecular Evolutionary Genetics Analysis Version 7.0 for Bigger Datasets. Mol Biol Evol.

[24] Felsenstein J (1985). Confidence limits on phylogenies: an approach using the bootstrap. Evolution.

[25] Hall TA (1999). BioEdit: a user-friendly biological sequence alignment editor and analysis program for windows 95/98/NT. Nucleic Acids Symp Ser.

[26] Sarwar M, Kremer RJ (1995). Determination of bacterially derived auxins using a microplate method. Lett Appl Microbiol.

[27] Beneduzi A, Peres D, Vargas LK, Bodanese-Zanettini MH, Passaglia LMP (2008). Evaluation of genetic diversity and plant growth promoting activities of nitrogen-fixing bacilli isolated from rice fields in South Brazil. Appl Soil Ecol.

[28] Bremner JM, Mulvaney CS, Page AL, Miller RH, Keeney DR (1982). Methods of Soil Analysis, Part 2: Chemical and Microbiological Properties.

[29] Sylvester-Bradley R, Asakawa N, Torraca SL, Magalhães FMM, Oliveira LA (1982). Levantamento quantitativo de microrganismos solubilizadores de fosfatos na rizosfera de gramíneas e leguminosas forrageiras na Amazônia. Acta Amaz.

[30] Schwyn B, Neilands JB (1987). Universal chemical assay for the detection and determination of siderophores. Anal Biochem.

[31] Hoagland DR, Arnon DI (1950). The Water-Culture Method for Growing Plants without Soil. 2nd ed.

[32] Coil D, Jospin G, Darling AE (2015). A5-miseq: an updated pipeline to assemble microbial genomes from illumina miseq data. Bioinformatics.

[33] Tatusova T, DiCuccio M, Badretdin A, Chetvernin V, Nawrocki EP (2016). NCBI prokaryotic genome annotation pipeline. Nucleic Acids Res.

[34] Asaf S, Khan AL, Khan MA, Al-Harrasi A, Lee IJ (2018). Complete genome sequencing and analysis of endophytic *Sphingomonas* sp. LK11 and its potential in plant growth. 3 Biotech.

[35] Yoon SH, Ha SM, Lim J, Kwon S, Chun J (2017). A large-scale evaluation of algorithms to calculate average nucleotide identity. Antonie Van Leeuwenhoek.

[36] Meier-Kolthoff JP, Auch AF, Klenk HP, Göker M (2013). Genome sequence-based species delimitation with confidence intervals and improved distance functions. BMC Bioinformatics.

[37] AleloMicro Consultas (2021). Empresa Brasileira de Pesquisa Agropecuária [Brazilian Agricultural Research Corporation] (Embrapa). https://am.cenargen.embrapa.br/amconsulta/especialidade?id=1.

[38] Fuentes-Ramirez LE, Jimenez-Salgado T, Abarca-Ocampo IR, Caballero-Mellado J (1993). *Acetobacter diazotrophicus*, an indoleacetic acid producing bacterium isolated from sugarcane cultivars of Mexico. Plant Soil.

[39] Sawana A, Adeolu M, Gupta RS (2014). Molecular signatures and phylogenomic analysis of the genus *Burkholderia*: proposal for division of this genus into the emended genus *Burkholderia* containing pathogenic organisms and a new genus *Paraburkholderia* gen. nov. harboring environmental species. Front Genet.

[40] Sfeir MM (2018). *Burkholderia cepacia* complex infections: More complex than the bacterium name suggest. J Infect.

[41] Rizi KS, Ghazvini K, Farsiani H (2019). Clinical and pathogens overview of enterobacter infections. Rev Clin Med.

[42] Kumar A, Rithesh L, Kumar V, Raghuvanshi N, Chaudhary K (2023). *Stenotrophomonas* in diversified cropping systems: friend or foe. Front Microbiol.

[43] Timilsina S, Potnis N, Newberry EA, Liyanapathiranage P, Iruegas-Bocardo F (2020). *Xanthomonas* diversity, virulence and plant–pathogen interactions. Nat Rev Microbiol.

[44] Chun J, Oren A, Ventosa A, Christensen H, Arahal DR (2018). Proposed minimal standards for the use of genome data for the taxonomy of prokaryotes. Int J Syst Evol Microbiol.

[45] Spaepen S, Vanderleyden J (2011). Auxin and plant-microbe interactions. Cold Spring Harb Perspect Biol.

[46] Shao J, Li S, Zhang N, Cui X, Zhou X (2015). Analysis and cloning of the synthetic pathway of the phytohormone indole-3-acetic acid in the plant-beneficial *Bacillus amyloliquefaciens* SQR9. Microb Cell Fact.

[47] Wanner BL (1993). Gene regulation by phosphate in enteric bacteria. J Cell Biochem.

[48] Rawat P, Das S, Shankhdhar D, Shankhdhar SC (2021). Phosphate-solubilizing microorganisms: mechanism and their role in phosphate solubilization and uptake. J Soil Sci Plant Nutr.

[49] Hartney SL, Mazurier S, Kidarsa TA, Quecine MC, Lemanceau P (2011). TonB-dependent outer-membrane proteins and siderophore utilization in *Pseudomonas fluorescens* Pf-5. Biometals.

[50] Zhao D, Curatti L, Rubio LM (2007). Evidence for *nifU* and *nifS* participation in the biosynthesis of the iron-molybdenum cofactor of nitrogenase. J Biol Chem.

