# Supplementary material for: Diversity and plant growth-promoting traits of endophytic bacteria isolated from maize cropped in organic and low-input agricultural systems in southern Brazil
Source: Access Microbiol. 2026 Jun 3;8(6):000948.v3. doi: 10.1099/acmi.0.000948.v3 (PMC13232961; doi:10.1099/acmi.0.000948.v3)
Supplement: Supplementary Material 1. [file acmi-8-00948-s001.pdf]

SUPPLEMENTARY MATERIAL

Diversity and plant growth-promoting traits of endophytic bacteria isolated from maize cropped in organic and low-input agricultural systems in southern Brazil

Dafila Santos Lima Fagotti, Josiane Fukami, Paula Cerezini, Andre Luiz Martinez Oliveira, Galdino Andrade, Milena Serenato Klepa, Luisa Carolina Ferraz Helene, Ligia Maria Oliveira Chueire, Renan Augusto Ribeiro, Mariangela Hungria, and Marco Antonio Nogueira

Table S1 Description of the sampling sites and characteristics of the soil (0-20 cm) in the sites cropped with maize sampled for isolating endophytic bacteria.

Description of sampling sites	1 and 2	3	4 and 5
Municipality/ State	Canoinhas/ SC	Porto União/ SC	São J. Triunfo/ PR
Coordinates	26°12' 55.92" S<br>50°34' 2.62" W	26°18' 23.48" S<br>51°10' 5.57" W	25°36' 13.32" S<br>50°22' 40.88" W
Maize landrace variety	Caiano	Fortuna	Amarelo Antigo
Soil management	No- tillage on vetch straw (1st yr of adoption)	No-tillage on oat and vetch straw (8 yr of adoption)	Conventional tillage
Fertilizers	Basalt powder (1 ton/ha)	Basalt powder (1 ton/ha)	No fertilizer
Agricultural system	Conventional to organic	Organic (3 yr of adoption)	Conventional with low-input
**Soil chemical analysis**			
Total C (g dm^{-3})	23.0	46.6	11.5
pH	4.88	4.74	4.30
Total N (g dm^{-3})	1.99	4.02	0.99
P (mg dm^{-3})	2.42	16.06	5.66
K ($cmol_c$ dm^{-3})	0.35	0.50	0.20
Ca ($cmol_c$ dm^{-3})	6.05	6.68	1.53
Mg ($cmol_c$ dm^{-3})	4.01	4.45	0.94
SB ($cmol_c$ dm^{-3})	10.41	11.63	2.67
H + Al ($cmol_c$ dm^{-3})	6.59	10.15	5.94
Al ($cmol_c$ dm^{-3})	0.00	0.16	0.85
CEC ($cmol_c$ dm^{-3})	17.01	21.78	8.61
V (%)	61.27	53.42	31.09
S-SO_4^{2-} (g dm^{-3})	2.27	6.81	2.89
B (mg dm^{-3})	0.60	1.03	0.78
Cu (mg dm^{-3})	1.60	4.92	0.81
Fe (mg dm^{-3})	58.05	52.49	149.9
Mn (mg dm^{-3})	27.35	16.33	25.45
Zn (mg dm^{-3})	0.01	1.14	1.15
**Granulometric fractions**			
Sand (g kg^{-1})	385	199	846
Silt (g kg^{-1})	134	281	43
Clay (g kg^{-1})	481	520	111

Table S2 Taxonomic identification, isolation site, culture medium of isolation, and GenBank accession numbers of 16S rRNA genes and genome sequences of 83 strains isolated, identified, and characterized for plant growth-promoting traits in this study.

Class	Identification	Isolation site [1]	Culture medium [2]	16S rRNA gene accession number
α-Proteobacteria (21 strains)	*Agrobacterium* CNPSo 2312	1	JNFb	JX174189
	Agrobacterium CNPSo 2327	2	JNFb	JX174204
	Agrobacterium CNPSo 2328	2	JNFb	JX174205
	Agrobacterium CNPSo 2329	2	JNFb	JX174206
	Agrobacterium CNPSo 2330	2	JNFb	JX174207
	Agrobacterium CNPSo 2331	2	JNFb	JX174208
	Agrobacterium CNPSo 2346	3	JNFb	JX174223
	Agrobacterium CNPSo 2347	3	JNFb	JX174224
	Agrobacterium CNPSo 2367	4	JNFb	JX174244
	Agrobacterium CNPSo 2368	4	JNFb	JX174245
	Agrobacterium CNPSo 2369	4	JNFb	JX174246
	Agrobacterium CNPSo 2382	5	JNFb	JX174259
	Rhizobium CNPSo 2320	1	JMV	JX174197
	Rhizobium CNPSo 2321	1	JMV	JX174198
	Rhizobium CNPSo 2322	1	JMV	JX174199
	Rhizobium CNPSo 2394	5	JMV	JX174271
	Rhizobium CNPSo 2395	5	LGI	JX174272
	Rhizobium CNPSo 2396	5	LGI	JX174273
	Sphingomonas CNPSo 2323	1	JNFb	JX174200
	Sphingomonas CNPSo 2363	3	JNFb	JX174240
	Sphingomonas CNPSo 2378	4	LGI	JX174255
β-Proteobacteria (26 strains)	*Burkholderia* CNPSo 2313	1	JMV	JX174190
	Burkholderia CNPSo 2314	1	JMV	JX174191
	Burkholderia CNPSo 2332	2	JMV	JX174209
	Burkholderia CNPSo 2333	2	JMV	JX174210
	Burkholderia CNPSo 2334	2	JMV	JX174211
	Burkholderia CNPSo 2337	2	LGI	JX174214
	Burkholderia CNPSo 2338	2	JMV	JX174215
	Burkholderia CNPSo 2339	2	JMV	JX174216
	Burkholderia CNPSo 2340	2	JMV	JX174217
	Burkholderia CNPSo 2348	3	LGI	JX174225
	Burkholderia CNPSo 2349	3	LGI	JX174226
	Burkholderia CNPSo 2350	3	LGI	JX174227
	Burkholderia CNPSo 2351	3	LGI	JX174228
	Burkholderia CNPSo 2352	3	JMV	JX174229
	Burkholderia CNPSo 2353	3	JMV	JX174230

Continue …

Table S2 Taxonomic identification, isolation site, culture medium of isolation, and GenBank accession numbers of 16S rRNA genes and genome sequences of 83 strains isolated, identified, and characterized for plant growth-promoting traits in this study.

**Class**	**Identification**	**Isolation site** [1]	**Culture medium** [2]	**16S rRNA gene accession number**
	Burkholderia CNPSo 2354	3	JMV	JX174231
	Burkholderia CNPSo 2371	4	JMV	JX174248
	Burkholderia CNPSo 2372	4	LGI	JX174249
	Burkholderia CNPSo 2387	5	LGI	JX174264
	Burkholderia CNPSo 2388	5	LGI	JX174265
	Burkholderia CNPSo 2389	5	LGI	JX174266
	Paraburkholderia CNPSo 2335	2	LGI	JX174212
	Paraburkholderia CNPSo 2336	2	LGI	JX174213
	Paraburkholderia CNPSo 2370	4	JMV	JX174247
	Paraburkholderia CNPSo 2385	5	JMV	JX174262
	Paraburkholderia CNPSo 2386	5	JMV	JX174263
γ-Proteobacteria (30 strains)	*Pseudomonas* CNPSo 2319	1	JNFb	JX174196
	Stenotrophomonas CNPSo 2324	1	JNFb	JX174201
	Stenotrophomonas CNPSo 2325	1	JNFb	JX174202
	Stenotrophomonas CNPSo 2326	1	JNFb	JX174203
	Stenotrophomonas CNPSo 2345	2	JNFb	JX174222
	Stenotrophomonas CNPSo 2365	3	JNFb	JX174242
	Stenotrophomonas CNPSo 2379	4	JNFb	JX174256
	Stenotrophomonas CNPSo 2380	4	JNFb	JX174257
	Stenotrophomonas CNPSo 2397	5	JNFb	JX174274
	Phytobacter CNPSo 2342	2	LGI	JX174219
	Phytobacter CNPSo 2344	2	JNFb	JX174221
	Phytobacter CNPSo 2356	3	LGI	JX174233
	Phytobacter CNPSo 2357	3	JNFb	JX174234
	Phytobacter CNPSo 2358	3	JNFb	JX174235
	Enterobacter CNPSo 2343	2	LGI	JX174220
	Enterobacter CNPSo 2373	4	LGI	JX174250
	Enterobacter CNPSo 2374	4	JMV	JX174251
	Enterobacter CNPSo 2375	4	JMV	JX174252
	Enterobacter CNPSo 2376	4	JNFb	JX174253
	Enterobacter CNPSo 2390	5	JNFb	JX174267
	Enterobacter CNPSo 2391	5	JNFb	JX174268
	Klebsiella CNPSo 2359	3	LGI	JX174236
	Klebsiella CNPSo 2392	5	JNFb	JX174269
	Pseudoxanthomonas CNPSo 2360	3	JMV	JX174237
	Pseudoxanthomonas CNPSo 2361	3	LGI	JX174238
	Pseudoxanthomonas CNPSo 2362	3	JNFb	JX174239

Continue …

Table S2 Taxonomic identification, isolation site, culture medium of isolation, and GenBank accession numbers of 16S rRNA genes and genome sequences of 83 strains isolated, identified, and characterized for plant growth-promoting traits in this study.

**Class**	**Identification**	**Isolation site** [1]	**Culture medium** [2]	**16S rRNA gene accession number**
	Pseudoxanthomonas CNPSo 2377	4	LGI	JX174254
	Pseudoxanthomonas CNPSo 2381	4	JMV	JX174258
	Pseudoxanthomonas CNPSo 2393	5	JNFb	JX174270
	Xanthomonas CNPSo 2366	3	JMV	JX174243
Actinomycetia (4 strains)	*Microbacterium* CNPSo 2315	1	JNFb	JX174192
	Microbacterium CNPSo 2316	1	JNFb	JX174193
	Microbacterium CNPSo 2317	1	JNFb	JX174194
	Microbacterium CNPSo 2318	1	JNFb	JX174195
Bacilli (2 strains)	*Bacillus* CNPSo 2383	5	JMV	JX174260
	Bacillus CNPSo 2384	5	JMV	JX174261

[1] According to Table S1. [2] The isolation procedure in different culture media has been described in the Material and Methods section.

Table S3 Characterization of endophytic bacteria isolated from maize for production of indolic compounds (estimated as IAA equivalent) with (+) and without (-) tryptophan, N concentration in N-free culture medium after seven days of incubation (Putative BNF), P-solubilization (tricalcium phosphate), and production of siderophores.

Taxonomic identification based on 16 rRNA analysis	IAA (+Tryp)	IAA (-Tryp)	Putative BNF	P-solubilization	Siderophores
	(μg mL^{-1})			Diameter of haloes (mm)	
Agrobacterium CNPSo 2312	2.94 ± 0.01	1.16	9.8	0	0
Agrobacterium CNPSo 2327	7.44 ± 0.20	0.60	7.0	0	0
Agrobacterium CNPSo 2328	3.43 ± 0.06	0.70	9.8	0	4.2 ± 0.2
Agrobacterium CNPSo 2329	1.73 ± 0.14	0.91	7.0	0	0
Agrobacterium CNPSo 2330	3.16 ± 0.10	1.93	12.6	0	42.3 ± 1.9
Agrobacterium CNPSo 2331	5.51 ± 0.22	0.46	8.4	0	5.5 ± 0.9
Agrobacterium CNPSo 2346	6.53 ± 1.21	1.33	12.6	0	1.0 ± 0.6
Agrobacterium CNPSo 2347	1.62 ± 0.11	0.53	7.0	0	4.3 ± 0.9
Agrobacterium CNPSo 2367	3.16 ± 0.32	0.46	16.8	0	9.7 ± 2.0
Agrobacterium CNPSo 2368	1.62 ± 0.10	0.56	8.4	0	10.7 ± 4.6
Agrobacterium CNPSo 2369	1.40 ± 0.15	0.63	14.0	0	8.3 ± 1.6
Agrobacterium CNPSo 2382	1.11 ± 0.10	0.11	11.2	0	11.1 ± 1.0
Rhizobium CNPSo 2320	1.32 ± 0.28	0.07	25.2	0	12.4 ± 0.6
Rhizobium CNPSo 2321	3.82 ± 1.46	0.49	15.4	0	14.0 ± 3.4
Rhizobium CNPSo 2322	2.61 ± 0.07	0.60	15.4	0	0
Rhizobium CNPSo 2394	3.92 ± 0.70	0.46	21.0	0	28.0 ± 2.0
Rhizobium CNPSo 2395	4.87 ± 0.27	0.60	11.2	0	11.4 ± 1.5
Rhizobium CNPSo 2396	3.69 ± 0.37	0.60	12.6	0	24.2 ± 4.0
Sphingomonas CNPSo 2323	3.03 ± 0.21	1.02	12.6	0	0
Sphingomonas CNPSo 2363	1.81 ± 0.16	0.81	9.8	0	0
Sphingomonas CNPSo 2378	5.85 ± 2.29	2.63	12.6	0	10 ± 1.4
Burkholderia CNPSo 2313	1.13 ± 0.32	2.21	14.0	11.2 ± 0.2	48.4 ± 0.4
Burkholderia CNPSo 2314	1.60 ± 0.80	2.73	14.0	4.3 ± 0.5	22.3 ± 1.1
Burkholderia CNPSo 2332	3.09 ± 0.10	0.91	29.4	5.3 ± 0.4	3.2 ± 0.7
Burkholderia CNPSo 2333	0.82 ± 0.17	0.46	16.8	4.3 ± 1.3	1.7 ± 1.0
Burkholderia CNPSo 2334	15.67 ± 0.42	1.23	16.8	0	0
Burkholderia CNPSo 2337	1.26 ± 0.43	2.31	15.4	9.0 ± 0.2	28.7 ± 5.0
Burkholderia CNPSo 2338	0.50 ± 0.02	0.21	16.8	9.3 ± 3.5	29.3 ± 2.3
Burkholderia CNPSo 2339	1.72 ± 0.99	2.49	15.4	8.9 ± 0.4	0
Burkholderia CNPSo 2340	1.44 ± 0.01	1.05	30.8	1.8 ± 0.6	27.0 ± 1.0
Burkholderia CNPSo 2348	1.09 ± 0.22	0.39	14.0	6.9 ± 1.1	0.4 ± 0.2
Burkholderia CNPSo 2349	3.57 ± 0.29	2.00	15.4	4.5 ± 0.8	0.7 ± 0.5
Burkholderia CNPSo 2350	1.27 ± 0.34	1.23	11.2	5.8 ± 0.1	44.0 ± 3.4
Burkholderia CNPSo 2351	0.67 ± 0.10	0.25	11.2	7.4 ± 0.9	12.0 ± 1.0
Burkholderia CNPSo 2352	1.02 ± 0.27	0.11	16.8	0	43.5 ± 1.8
Burkholderia CNPSo 2353	3.20 ± 0.02	3.61	12.6	7.8 ± 3.2	27.0 ± 1.6

Continue …

Table S3 Characterization of endophytic bacteria isolated from maize for production of indolic compounds (estimated as IAA equivalent) with (+) and without (-) tryptophan, N concentration in N-free culture medium after seven days of incubation (Putative BNF), P-solubilization (tricalcium phosphate), and production of siderophores.

Taxonomic identification based on 16 rRNA analysis	IAA (+Tryp)	IAA (-Tryp)	Putative BNF	P-solubilization	Siderophores
		($\mu g\ mL^{-1}$)		Diameter of haloes (mm)	
Burkholderia CNPSo 2354	0.34 ± 0.08	0.07	15.4	7.8 ± 0.6	16.7 ± 2.2
Burkholderia CNPSo 2371	0.51 ± 0.25	0.14	16.8	6.0 ± 0.3	27.2 ± 6.0
Burkholderia CNPSo 2372	0.56 ± 0.12	0.18	14.0	10.1 ± 0.2	30.3 ± 3.6
Burkholderia CNPSo 2387	0.84 ± 0.17	0.28	11.2	7.2 ± 0.2	23.5 ± 0.6
Burkholderia CNPSo 2388	0.26 ± 0.05	0.21	15.4	3.8 ± 0.2	10.2 ± 0.3
Burkholderia CNPSo 2389	1.67 ± 0.47	1.93	12.6	8.1 ± 0.4	36.1 ± 2.7
Paraburkholderia CNPSo 2335	0.78 ± 0.29	1.19	9.8	9.0 ± 0.4	17.9 ± 1.2
Paraburkholderia CNPSo 2336	0.88 ± 0.16	0.95	11.2	6.3 ± 1.4	25.7 ± 1.1
Paraburkholderia CNPSo 2370	0.75 ± 0.16	1.16	14.0	0	18.4 ± 2.1
Paraburkholderia CNPSo 2385	0.64 ± 0.08	0.21	23.8	0	39.7 ± 1.7
Paraburkholderia CNPSo 2386	2.14 ± 0.34	0.35	19.6	0	7.9 ± 1.1
Pseudomonas CNPSo 2319	1.18 ± 0.29	0.77	11.2	0	0
Stenotrophomonas CNPSo 2324	1.46 ± 0.73	0.39	12.6	0	4.3 ± 0.4
Stenotrophomonas CNPSo 2325	0.09 ± 0.02	0.11	12.0	0	0
Stenotrophomonas CNPSo 2326	15.49 ± 1.10	6.44	7.0	0	0
Stenotrophomonas CNPSo 2345	4.25 ± 0.65	0.32	8.4	0	10.3 ± 1.3
Stenotrophomonas CNPSo 2365	1.20 ± 0.35	1.37	11.2	0	0.8 ± 0.2
Stenotrophomonas CNPSo 2379	1.12 ± 0.19	0.18	15.4	0	8.1 ± 1.6
Stenotrophomonas CNPSo 2380	0.61 ± 0.10	0.07	15.4	0	10.2 ± 0.2
Stenotrophomonas CNPSo 2397	1.38 ± 0.38	0.42	8.4	0	2.5 ± 0.8
Phytobacter CNPSo 2342	1.12 ± 0.07	1.40	12.6	0	0
Phytobacter CNPSo 2344	5.30 ± 0.63	0.39	12.6	0	2.8 ± 0.4
Phytobacter CNPSo 2356	1.06 ± 0.08	0.35	15.4	0	0
Phytobacter CNPSo 2357	1.30 ± 0.39	0.53	7.0	0	8.7 ± 1.1
Phytobacter CNPSo 2358	1.82 ± 0.04	0.81	9.8	0	5.6 ± 0.6
Enterobacter CNPSo 2343	20.37 ± 1.88	3.05	9.8	0	8.2 ± 0.2
Enterobacter CNPSo 2373	2.47 ± 0.02	2.38	9.8	0	2.9 ± 0.9
Enterobacter CNPSo 2374	10.36 ± 0.55	2.35	18.2	0	33.1 ± 2.7
Enterobacter CNPSo 2375	2.89 ± 0.35	2.45	19.6	0	17.7 ± 1.5
Enterobacter CNPSo 2376	11.90 ± 0.71	2.70	5.6	0	3.7 ± 0.3
Enterobacter CNPSo 2390	2.81 ± 0.17	2.14	8.4	0	33.7 ± 3.8
Enterobacter CNPSo 2391	26.95 ± 2.80	2.24	8.4	0	10.9 ± 2.2
Klebsiella CNPSo 2359	7.46 ± 0.42	0.18	16.8	0	0
Klebsiella CNPSo 2392	2.00 ± 0.12	0.28	15.4	0	0
Pseudoxanthomonas CNPSo 2360	1.72 ± 0.07	0.32	12.6	0	4.2 ± 0.2

Continue …

Table S3 Characterization of endophytic bacteria isolated from maize for production of indolic compounds (estimated as IAA equivalent) with (+) and without (-) tryptophan, N concentration in N-free culture medium after seven days of incubation (Putative BNF), P-solubilization (tricalcium phosphate), and production of siderophores.

Taxonomic identification based on 16 rRNA analysis	IAA (+Tryp)	IAA (-Tryp)	Putative BNF	P-solubilization	Siderophores
		($\mu g\ mL^{-1}$)		Diameter of haloes (mm)	
Pseudoxanthomonas CNPSo 2361	4.12 ± 0.19	0.53	12.6	0	0
Pseudoxanthomonas CNPSo 2362	2.51 ± 0.05	0.07	9.8	0	4.2 ± 1.1
Pseudoxanthomonas CNPSo 2377	3.17 ± 0.27	0.18	12.6	0	0
Pseudoxanthomonas CNPSo 2381	1.46 ± 0.28	1.05	16.8	0	15.9 ± 2.2
Pseudoxanthomonas CNPSo 2393	6.06 ± 0.14	3.40	11.2	0	17.1 ± 2.4
Xanthomonas CNPSo 2366	2.53 ± 0.60	2.21	12.6	0	41.5 ± 4.7
Microbacterium CNPSo 2315	0.25 ± 0.12	0.35	12.6	0	0
Microbacterium CNPSo 2316	0.09 ± 0.11	0.42	8.4	0	0
Microbacterium CNPSo 2317	0.09 ± 0.04	0.18	12.6	0	0
Microbacterium CNPSo 2318	0.46 ± 0.16	0.32	7.0	0	0
Bacillus CNPSo 2383	0.99 ± 0.28	0.56	12.6	0	0
Bacillus CNPSo 2384	1.74 ± 0.10	4.13	11.2	0	17.0 ± 1.0
Azospirillum brasilense Ab-V5	16.04 ± 2.72	0.91	-	0	0

Table S4 Genomic parameters of the strains *Sphingomonas* sp. CNPSo 2378 and *Bacillus velezensis* CNPSo 2384.

**Genomic statistics**	**Strains**	
**Statistics of genome assemblies**	***Sphingomonas* sp. CNPSo 2378**	***Bacillus velezensis* CNPSo 2384**
Size (bp)	4,331,468	4,133,224
No. of contigs	184	74
N50	77,510	194,138
Coverage	381×	415×
G+C content (%)	66.43	46.02
No. of coding sequences	3,843	4,051
Pseudogenes	66	89
No. of RNAs	61	95
**Genomic comparisons**		
**Closest strains**	**orthoANIu (%)**	**dHDD (%)**
	***Sphingomonas* sp. CNPSo 2378**	
S. sanguinis NBRC 13937	86.1	31.1
S. parapaucimobilis NBRC 15100	77.39	31.2
	***Bacillus velezensis* CNPSo 2384**	
B. velezensis subsp. *plantarum* FZB42 (= *B. amyloliquefasciens*)	98.93	90.6
B. velezensis NRRL-B41580	98.37	85
B. velezensis KACC 13105 (= *B. methylotrophicus*)	98.26	84.4
B. siamensis KCTC 13613	94.35	56.4
B. amyloliquefasciens DSM 7	94.04	55.2
B. tequilensis ATCC BAA-819	77.39	20.7
B. subtilis NBRC 13719	77.03	31.5

Table S5 Putative genes involved in plant growth-promotion traits and pathogen suppression in the genome of the strains *Sphingomonas* sp. CNPSo 2378 and *Bacillus velezensis* CNPSo 2384.

	***Sphingomonas* sp. CNPSo 2374**		
**Function**	**Gene**	**Protein**	**Locus_tag**
IAA biosynthesis	*trpA*	tryptophan synthase subunit alpha	SM191_08050
	trpB	tryptophan synthase subunit beta	SM191_08055
	trpC	indole-3-glycerol phosphate synthase TrpC	SM191_03465
	trpD	anthranilate phosphoribosyltransferase	SM191_03460
	trpE	anthranilate synthase component I	SM191_03445
BNF	*nifU*	NifU family protein	SM191_16435
Sidererophore		TonB-dependent receptor	102 genes
Phosphate solubilization	*phoB*	phosphate regulon transcriptional regulator	SM191_07705
	phoU	phosphate signaling complex protein PhoU	SM191_07710
	phoR	HAMP domain-containing sensor histidine kinase	SM191_17325
	phoR	ATP-binding protein	SM191_07735
	pstA	phosphate ABC transporter permease PstA	SM191_07720
	pstB	phosphate ABC transporter ATP-binding protein PstB	SM191_07715
	pstC	phosphate ABC transporter permease subunit PstC	SM191_07725
	pstS	substrate-binding domain-containing protein	SM191_07730
	***Bacillus velezensis* CNPSo 2384**		
**Function**	**Gene**	**Protein**	**Locus_tag**
IAA biosynthesis	*ysnE*	GNAT family N-acetyltransferase	SM193_04295
	yclB	non-oxidative hydroxyarylic acid decarboxylases subunit B	SM193_12850
	yclC	phenolic acid decarboxylase BsdC	SM193_12845
	yhcX	bifunctional GNAT family N-acetyltransferase/carbon-nitrogen hydrolase family	SM193_10205
	dhaS	aldehyde dehydrogenase family protein	SM193_18350
	patB	MalY/PatB family protein	SM193_15945
	trpA	tryptophan synthase subunit alpha	SM193_08605
	trpB	tryptophan synthase subunit beta	SM193_08600
	trpC	indole-3-glycerol phosphate synthase TrpC	SM193_08590
	trpD	anthranilate phosphoribosyltransferase	SM193_08585
	trpE	anthranilate synthase component I	SM193_08580
BNF	*nifU*	NifU family protein	SM193_15540
Phosphate solubilization	*pstA*	phosphate ABC transporter permease PstA	SM193_14140
	pstB	phosphate ABC transporter ATP-binding protein PstB	SM193_14145
	pstC	phosphate ABC transporter permease subunit PstC	SM193_14135
	phoH	PhoH family protein	SM193_02155

Continue…

Table S5 Putative genes involved in plant growth-promotion traits and pathogen suppression in the genome of the strains *Sphingomonas* sp. CNPSo 2378 and *Bacillus velezensis* CNPSo 2384.

***Sphingomonas* sp. CNPSo 2374**			
**Function**	**Gene**	**Protein**	**Locus_tag**
	phyC	3-phytase	SM193_18055
Bacillibactin (siderophore)	*dhbA*	2,3-dihydro-2,3-dihydroxybenzoate dehydrogenase	SM193_15680
	dhbB	isochorismatase	SM193_15695
	dhbC	isochorismate synthase DhbC	SM193_15685
	dhbE	(2,3-dihydroxybenzoyl)adenylate synthase	SM193_15690
	dhbF	amino acid adenylation domain-containing protein	SM193_15700
Surfactin (biofilm, induction of ISR)	*srfAC*	surfactin non-ribosomal peptide synthetase	SM193_12910
	srfAD	surfactin biosynthesis thioesterase	SM193_12905
	srfAA	surfactin non-ribosomal peptide synthetase	SM193_12920
	srfAB	surfactin non-ribosomal peptide synthetase	SM193_20545
Fengycin (induction of ISR)	*fenA*	condensation domain-containing protein	SM193_20180
	fenB	non-ribosomal peptide synthetase	SM193_20520
	fenC	condensation domain-containing protein	SM193_20525
	fenD	amino acid adenylation domain-containing protein	SM193_20535
	fenE	non-ribosomal peptide synthetase	SM193_12030
Bacillomycin-D (induction of ISR)	*bmyA*	bacillomycin D hybrid PKS/NRPS BamA	SM193_11910
	bmyB	non-ribosomal peptide synthase/polyketide synthase	SM193_11905
	bmyC	bacillomycin D non-ribosomal peptide synthetase bamC	SM193_11900
	bmyD	bacillomycin D biosynthesis malonyl-CoA transacylase BamD	SM193_11915
Difficidin (direct suppression of bacteria)	*dfnM*	polyketide synthase	SM193_19925
	dfnL	hydroxymethylglutaryl-CoA synthase family protein	SM193_19930
	dfnK	cytochrome P450	SM193_19935
	dfnJ	beta-ketoacyl synthase N-terminal-like domain containing protein	SM193_19940
	dfnI	type I polyketide synthase	SM193_19945
	dfnH	type I polyketide synthase	SM193_19950
	dfnG	beta-ketoacyl synthase N-terminal-like domain containing protein	SM193_19955
	dfnG	SDR family NAD(P)-dependent oxidoreductase	SM193_20350
	dfnF	type I polyketide synthase	SM193_20355
	dfnE	type I polyketide synthase	SM193_20360
	dfnD	SDR family NAD(P)-dependent oxidoreductase	SM193_20510

Continue…

Table S5 Putative genes involved in plant growth-promotion traits and pathogen suppression in the genome of the strains *Sphingomonas* sp. CNPSo 2378 and *Bacillus velezensis* CNPSo 2384.

***Sphingomonas* sp. CNPSo 2374**			
**Function**	**Gene**	**Protein**	**Locus_tag**
	dfnC	SDR family oxidoreductase	SM193_20505
	dfnX	acyl carrier protein	SM193_20495
	dfnY	D-fructose-6-phosphate amidotransferase	SM193_20490
	dfnA	ACP S-malonyltransferase	SM193_20485
Bacillaenae (direct suppression of bacteria)	*baeB*	MBL fold metallo-hydrolase	SM193_03275
	baeC	ACP S-malonyltransferase	SM193_03280
	baeD	acyltransferase domain-containing protein	SM193_03285
	baeE	ACP S-malonyltransferase	SM193_03290
	acpK	acyl carrier protein	SM193_03295
	baeG	hydroxymethylglutaryl-CoA synthase family protein	SM193_03300
	baeH	enoyl-CoA hydratase/isomerase	SM193_03305
	baeI	polyketide synthase	SM193_03310
	baeJ	non-ribosomal peptide synthetase	SM193_03315
	baeL	SDR family NAD(P)-dependent oxidoreductase	SM193_03320
	baeM	SDR family NAD(P)-dependent oxidoreductase	SM193_03325
	baeN	amino acid adenylation domain-containing protein	SM193_03330
	baeR	beta-ketoacyl synthase N-terminal-like domain containing protein	SM193_03335
	baeS	cytochrome P450	SM193_03340
Macrolactin (direct suppression of bacteria)	*mlnA*	ACP S-malonyltransferase	SM193_02000
	mlnB	SDR family NAD(P)-dependent oxidoreductase	SM193_02005
	mlnC	polyketide synthase of type I	SM193_02010
	mlnD	polyketide synthase of type I	SM193_02015
	mlnE	SDR family NAD(P)-dependent oxidoreductase	SM193_02020
	mlnF	SDR family NAD(P)-dependent oxidoreductase	SM193_02025
	mlnG	SDR family NAD(P)-dependent oxidoreductase	SM193_02030
	mlnH	alpha/beta fold hydrolase	SM193_02035
	mlnI	serine hydrolase domain-containing protein	SM193_02040
Bacilysin (direct suppression of bacteria and cyanobacteria)	*bacA*	bacilysin biosynthesis protein BacA	SM193_04485
	bacB	cupin domain-containing protein	SM193_04490
	bacC	dihydroanticapsin 7-dehydrogenase	SM193_04495
	bacD	ATP-grasp domain-containing protein	SM193_04500
	bacE	MFS transporter	SM193_04505
	ywfG	pyridoxal phosphate-dependent aminotransferase	SM193_04510

Legends of Supplementary Figures

Fig. S1 Maximum likelihood phylogeny based on the 16S rRNA gene alignment (1,261 bp), using the Tamura 3-parameter model +G+I by MEGA v. 7. Accession numbers are indicated in parentheses and in Table S2. The α-Proteobacteria strains isolated in this study are shown in bold, and the promising strains are marked with a maize cob image. Bootstrap values >70 % are indicated at the nodes. Bar indicates the percentage of nucleotide substitutions.

Fig. S2 Maximum likelihood phylogeny based on the 16S rRNA gene alignment (1,275 bp), using the Hasegawa-Kishino-Yano model +G+I by MEGA v. 7. Accession numbers are indicated in parentheses and in Table S2. The β-Proteobacteria strains isolated in this study are shown in bold, and the promising strains are marked with a maize cob image. Bootstrap values >70 % are indicated at the nodes. Bar indicates the percentage of nucleotide substitutions.

Fig. S3 Maximum likelihood phylogeny based on the 16S rRNA gene alignment (1,275 bp), using the Tamura-Nei model +G by MEGA v. 7. Accession numbers are indicated in parentheses and in Table S2. The γ-Proteobacteria strains isolated in this study are shown in bold, and the promising strains are marked with a maize cob image. Bootstrap values >70 % are indicated at the nodes. Bar indicates the percentage of nucleotide substitutions.

Fig. S4 Maximum likelihood phylogeny based on the 16S rRNA gene alignment (1,325 bp), using the Tamura 3-parameter model +G+I by MEGA v. 7. Accession numbers are indicated in parentheses and in Table S2. The Actinomycetia strains isolated in this study are shown in bold, and the promising strains are marked with a maize cob image. Bootstrap values >70 % are indicated at the nodes. Bar indicates the percentage of nucleotide substitutions.

Fig. S5 Maximum likelihood phylogeny based on the 16S rRNA gene alignment (1,307 bp), using the Hasegawa-Kishino-Yano model +G by MEGA v. 7. Accession numbers are indicated in parentheses and in Table S2. The Bacilli strains isolated in this study are shown in bold, and the promising strains are marked with a maize cob image. Bootstrap values >70 % are indicated at the nodes. Bar indicates the percentage of nucleotide substitutions.

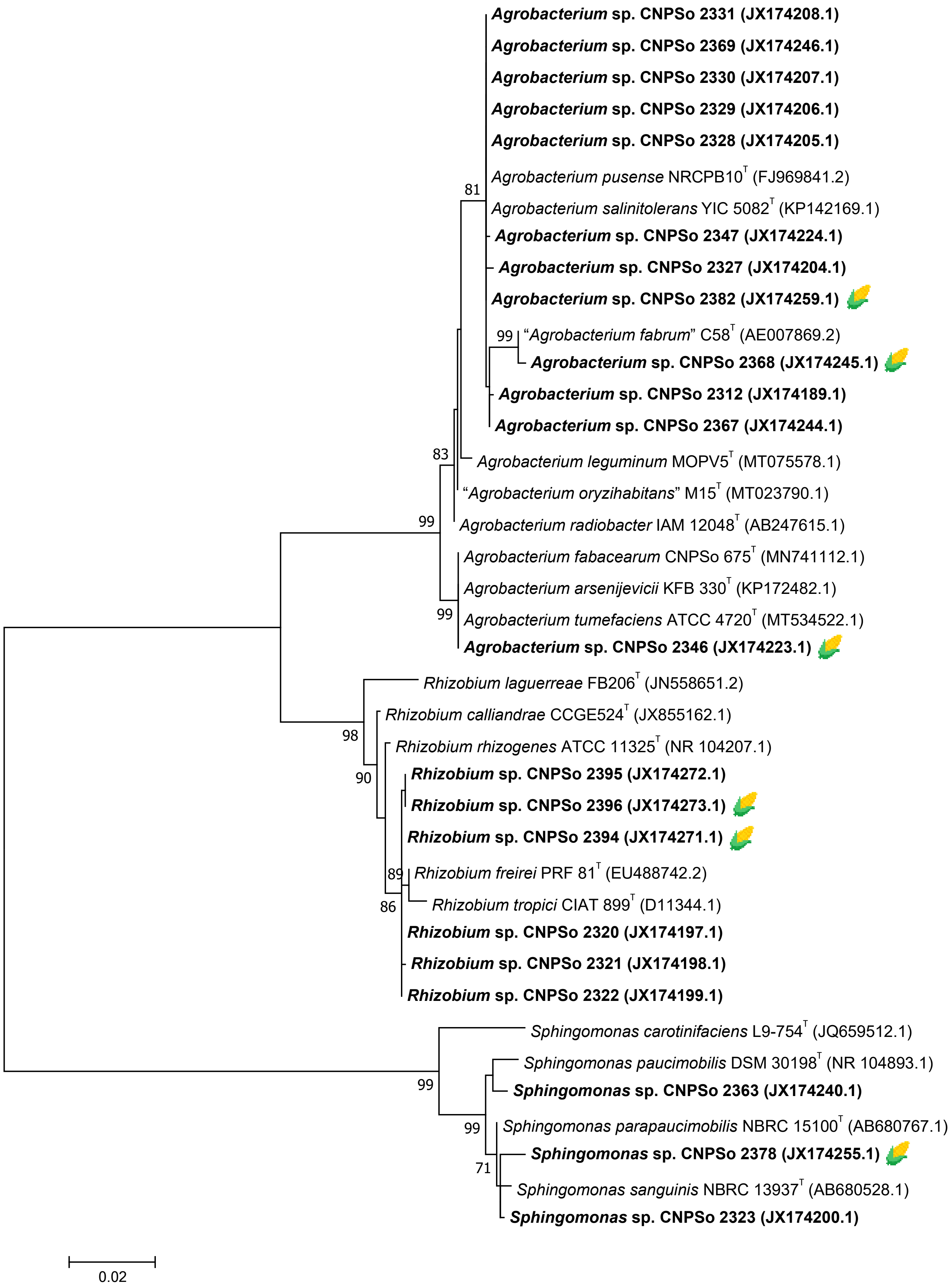

Agrobacterium sp. CNPSo 2331 (JX174208.1)
Agrobacterium sp. CNPSo 2369 (JX174246.1)
Agrobacterium sp. CNPSo 2330 (JX174207.1)
Agrobacterium sp. CNPSo 2329 (JX174206.1)
Agrobacterium sp. CNPSo 2328 (JX174205.1)
Agrobacterium pusense NRCPB10T (FJ969841.2)
81
Agrobacterium salinitolerans YIC 5082T (KP142169.1)
Agrobacterium sp. CNPSo 2347 (JX174224.1)
Agrobacterium sp. CNPSo 2327 (JX174204.1)
Agrobacterium sp. CNPSo 2382 (JX174259.1)
99
"Agrobacterium fabrum" C58T (AE007869.2)
Agrobacterium sp. CNPSo 2368 (JX174245.1)
Agrobacterium sp. CNPSo 2312 (JX174189.1)
Agrobacterium sp. CNPSo 2367 (JX174244.1)
83
Agrobacterium leguminum MOPV5T (MT075578.1)
"Agrobacterium oryzihabitans" M15T (MT023790.1)
99
Agrobacterium radiobacter IAM 12048T (AB247615.1)
Agrobacterium fabacearum CNPSo 675T (MN741112.1)
Agrobacterium arsenijevicii KFB 330T (KP172482.1)
99
Agrobacterium tumefaciens ATCC 4720T (MT534522.1)
Agrobacterium sp. CNPSo 2346 (JX174223.1)
Rhizobium laguerreae FB206T (JN558651.2)
Rhizobium calliandrae CCGE524T (JX855162.1)
98
Rhizobium rhizogenes ATCC 11325T (NR 104207.1)
90
Rhizobium sp. CNPSo 2395 (JX174272.1)
Rhizobium sp. CNPSo 2396 (JX174273.1)
Rhizobium sp. CNPSo 2394 (JX174271.1)
89
Rhizobium freirei PRF 81T (EU488742.2)
86
Rhizobium tropici CIAT 899T (D11344.1)
Rhizobium sp. CNPSo 2320 (JX174197.1)
Rhizobium sp. CNPSo 2321 (JX174198.1)
Rhizobium sp. CNPSo 2322 (JX174199.1)
Sphingomonas carotinifaciens L9-754T (JQ659512.1)
Sphingomonas paucimobilis DSM 30198T (NR 104893.1)
99
Sphingomonas sp. CNPSo 2363 (JX174240.1)
99
Sphingomonas parapaucimobilis NBRC 15100T (AB680767.1)
Sphingomonas sp. CNPSo 2378 (JX174255.1)
71
Sphingomonas sanguinis NBRC 13937T (AB680528.1)
Sphingomonas sp. CNPSo 2323 (JX174200.1)
0.02

Fig. S1

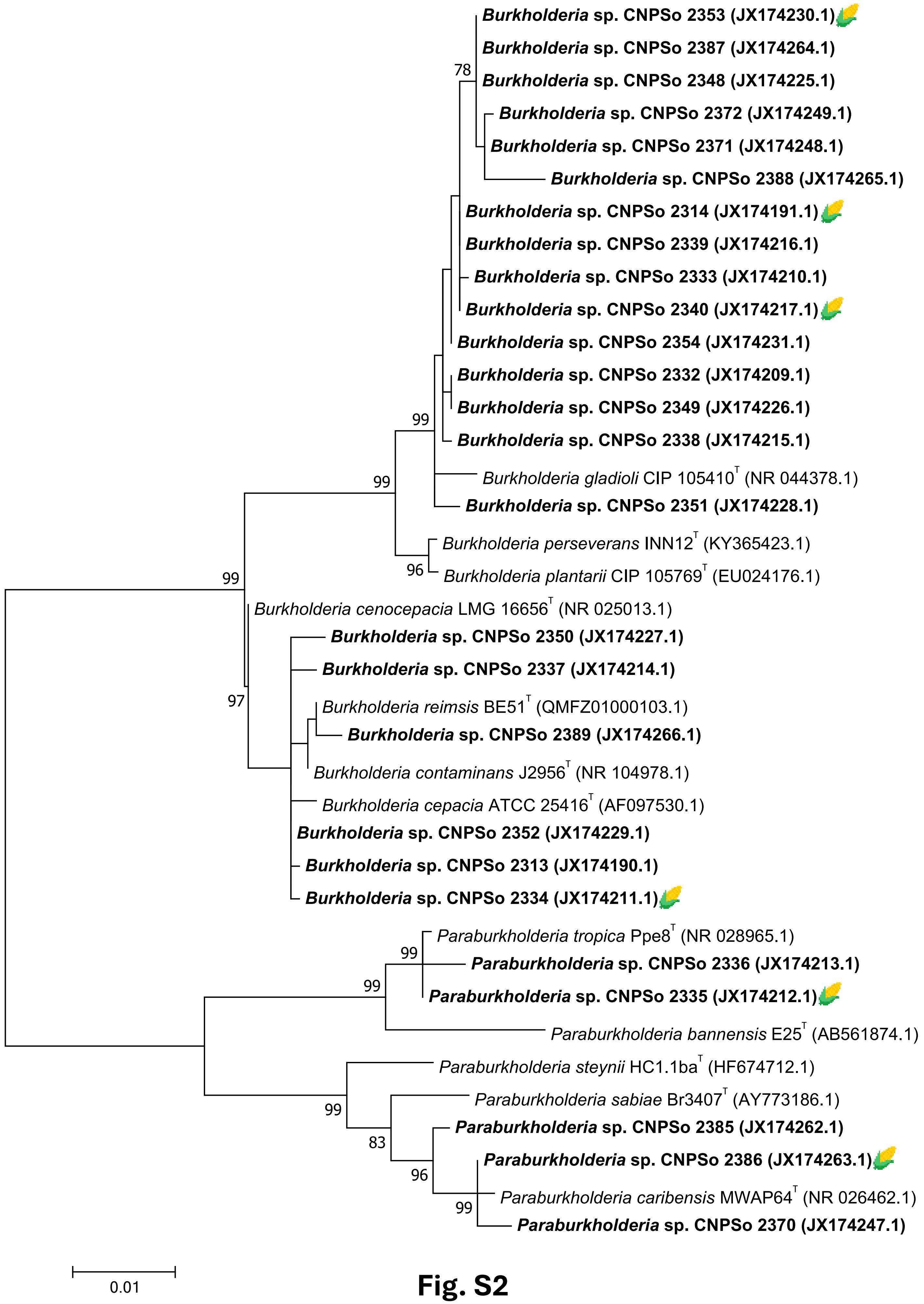
Burkholderia sp. CNPSo 2353 (JX174230.1)
Burkholderia sp. CNPSo 2387 (JX174264.1)
Burkholderia sp. CNPSo 2348 (JX174225.1)
Burkholderia sp. CNPSo 2372 (JX174249.1)
Burkholderia sp. CNPSo 2371 (JX174248.1)
Burkholderia sp. CNPSo 2388 (JX174265.1)
Burkholderia sp. CNPSo 2314 (JX174191.1)
Burkholderia sp. CNPSo 2339 (JX174216.1)
Burkholderia sp. CNPSo 2333 (JX174210.1)
Burkholderia sp. CNPSo 2340 (JX174217.1)
Burkholderia sp. CNPSo 2354 (JX174231.1)
Burkholderia sp. CNPSo 2332 (JX174209.1)
Burkholderia sp. CNPSo 2349 (JX174226.1)
Burkholderia sp. CNPSo 2338 (JX174215.1)
Burkholderia gladioli CIP 105410T (NR 044378.1)
Burkholderia sp. CNPSo 2351 (JX174228.1)
Burkholderia perseverans INN12T (KY365423.1)
Burkholderia plantarii CIP 105769T (EU024176.1)
Burkholderia cenocepacia LMG 16656T (NR 025013.1)
Burkholderia sp. CNPSo 2350 (JX174227.1)
Burkholderia sp. CNPSo 2337 (JX174214.1)
Burkholderia reimsis BE51T (QMFZ01000103.1)
Burkholderia sp. CNPSo 2389 (JX174266.1)
Burkholderia contaminans J2956T (NR 104978.1)
Burkholderia cepacia ATCC 25416T (AF097530.1)
Burkholderia sp. CNPSo 2352 (JX174229.1)
Burkholderia sp. CNPSo 2313 (JX174190.1)
Burkholderia sp. CNPSo 2334 (JX174211.1)
Paraburkholderia tropica Ppe8T (NR 028965.1)
Paraburkholderia sp. CNPSo 2336 (JX174213.1)
Paraburkholderia sp. CNPSo 2335 (JX174212.1)
Paraburkholderia bannensis E25T (AB561874.1)
Paraburkholderia steynii HC1.1baT (HF674712.1)
Paraburkholderia sabiae Br3407T (AY773186.1)
Paraburkholderia sp. CNPSo 2385 (JX174262.1)
Paraburkholderia sp. CNPSo 2386 (JX174263.1)
Paraburkholderia caribensis MWAP64T (NR 026462.1)
Paraburkholderia sp. CNPSo 2370 (JX174247.1)
78
99
99
99
96
97
99
99
99
83
96
99
0.01

Fig. S2

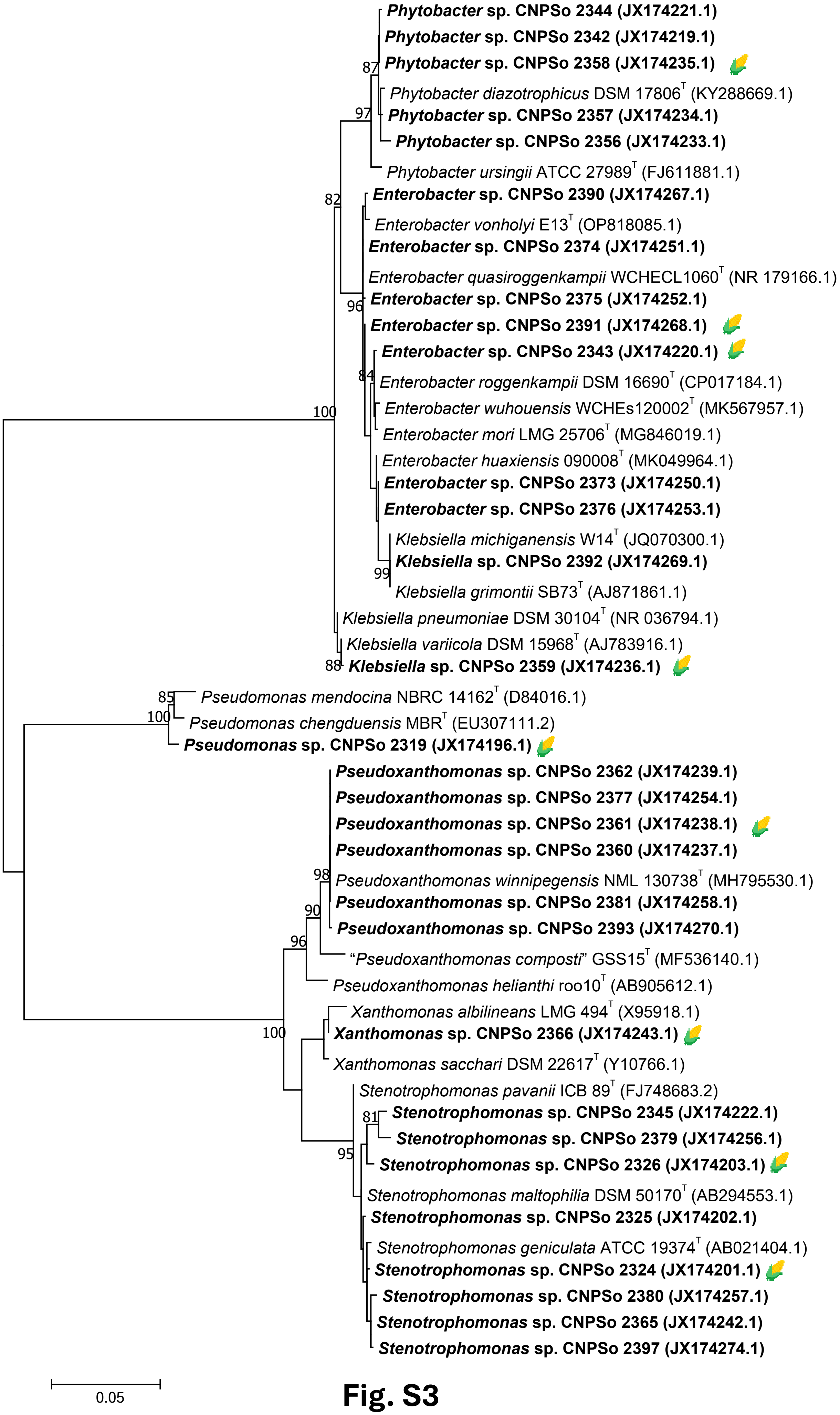
Phytobacter sp. CNPSo 2344 (JX174221.1)
Phytobacter sp. CNPSo 2342 (JX174219.1)
Phytobacter sp. CNPSo 2358 (JX174235.1)
Phytobacter diazotrophicus DSM 17806T (KY288669.1)
Phytobacter sp. CNPSo 2357 (JX174234.1)
Phytobacter sp. CNPSo 2356 (JX174233.1)
Phytobacter ursingii ATCC 27989T (FJ611881.1)
Enterobacter sp. CNPSo 2390 (JX174267.1)
Enterobacter vonholyi E13T (OP818085.1)
Enterobacter sp. CNPSo 2374 (JX174251.1)
Enterobacter quasiroggenkampii WCHECL1060T (NR 179166.1)
Enterobacter sp. CNPSo 2375 (JX174252.1)
Enterobacter sp. CNPSo 2391 (JX174268.1)
Enterobacter sp. CNPSo 2343 (JX174220.1)
Enterobacter roggenkampii DSM 16690T (CP017184.1)
Enterobacter wuhouensis WCHEs120002T (MK567957.1)
Enterobacter mori LMG 25706T (MG846019.1)
Enterobacter huaxiensis 090008T (MK049964.1)
Enterobacter sp. CNPSo 2373 (JX174250.1)
Enterobacter sp. CNPSo 2376 (JX174253.1)
Klebsiella michiganensis W14T (JQ070300.1)
Klebsiella sp. CNPSo 2392 (JX174269.1)
Klebsiella grimontii SB73T (AJ871861.1)
Klebsiella pneumoniae DSM 30104T (NR 036794.1)
Klebsiella variicola DSM 15968T (AJ783916.1)
Klebsiella sp. CNPSo 2359 (JX174236.1)
Pseudomonas mendocina NBRC 14162T (D84016.1)
Pseudomonas chengduensis MBRT (EU307111.2)
Pseudomonas sp. CNPSo 2319 (JX174196.1)
Pseudoxanthomonas sp. CNPSo 2362 (JX174239.1)
Pseudoxanthomonas sp. CNPSo 2377 (JX174254.1)
Pseudoxanthomonas sp. CNPSo 2361 (JX174238.1)
Pseudoxanthomonas sp. CNPSo 2360 (JX174237.1)
Pseudoxanthomonas winnipegensis NML 130738T (MH795530.1)
Pseudoxanthomonas sp. CNPSo 2381 (JX174258.1)
Pseudoxanthomonas sp. CNPSo 2393 (JX174270.1)
"Pseudoxanthomonas composti" GSS15T (MF536140.1)
Pseudoxanthomonas helianthi roo10T (AB905612.1)
Xanthomonas albilineans LMG 494T (X95918.1)
Xanthomonas sp. CNPSo 2366 (JX174243.1)
Xanthomonas sacchari DSM 22617T (Y10766.1)
Stenotrophomonas pavanii ICB 89T (FJ748683.2)
Stenotrophomonas sp. CNPSo 2345 (JX174222.1)
Stenotrophomonas sp. CNPSo 2379 (JX174256.1)
Stenotrophomonas sp. CNPSo 2326 (JX174203.1)
Stenotrophomonas maltophilia DSM 50170T (AB294553.1)
Stenotrophomonas sp. CNPSo 2325 (JX174202.1)
Stenotrophomonas geniculata ATCC 19374T (AB021404.1)
Stenotrophomonas sp. CNPSo 2324 (JX174201.1)
Stenotrophomonas sp. CNPSo 2380 (JX174257.1)
Stenotrophomonas sp. CNPSo 2365 (JX174242.1)
Stenotrophomonas sp. CNPSo 2397 (JX174274.1)
87
97
82
96
84
100
99
88
85
100
98
90
96
100
81
95
0.05

Fig. S3

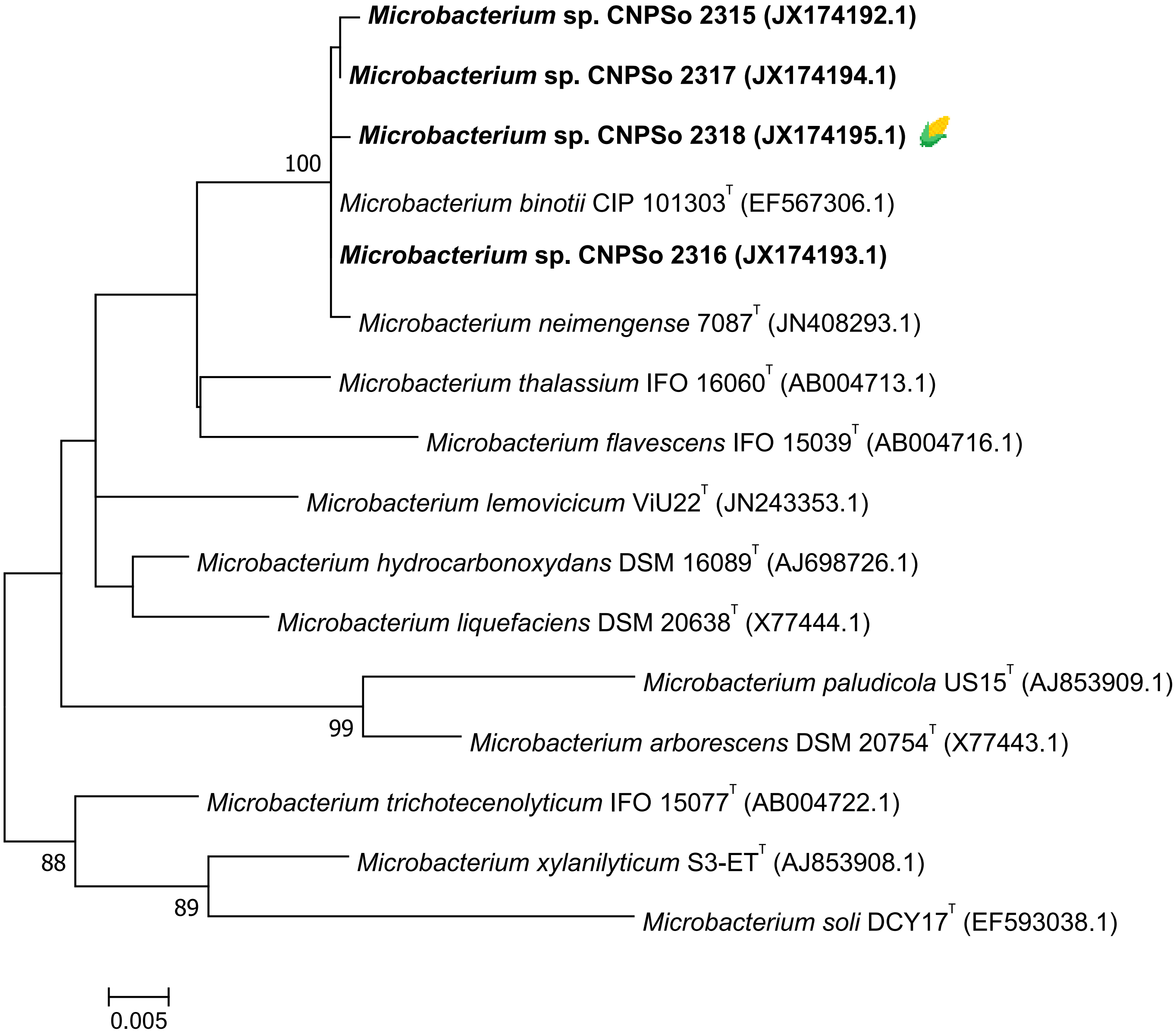
Microbacterium sp. CNPSo 2315 (JX174192.1)
Microbacterium sp. CNPSo 2317 (JX174194.1)
Microbacterium sp. CNPSo 2318 (JX174195.1)
100
Microbacterium binotii CIP 101303T (EF567306.1)
Microbacterium sp. CNPSo 2316 (JX174193.1)
Microbacterium neimengense 7087T (JN408293.1)
Microbacterium thalassium IFO 16060T (AB004713.1)
Microbacterium flavescens IFO 15039T (AB004716.1)
Microbacterium lemovicicum ViU22T (JN243353.1)
Microbacterium hydrocarbonoxydans DSM 16089T (AJ698726.1)
Microbacterium liquefaciens DSM 20638T (X77444.1)
Microbacterium paludicola US15T (AJ853909.1)
99
Microbacterium arborescens DSM 20754T (X77443.1)
Microbacterium trichotecenolyticum IFO 15077T (AB004722.1)
88
Microbacterium xylanilyticum S3-ETT (AJ853908.1)
89
Microbacterium soli DCY17T (EF593038.1)
0.005

Fig. S4

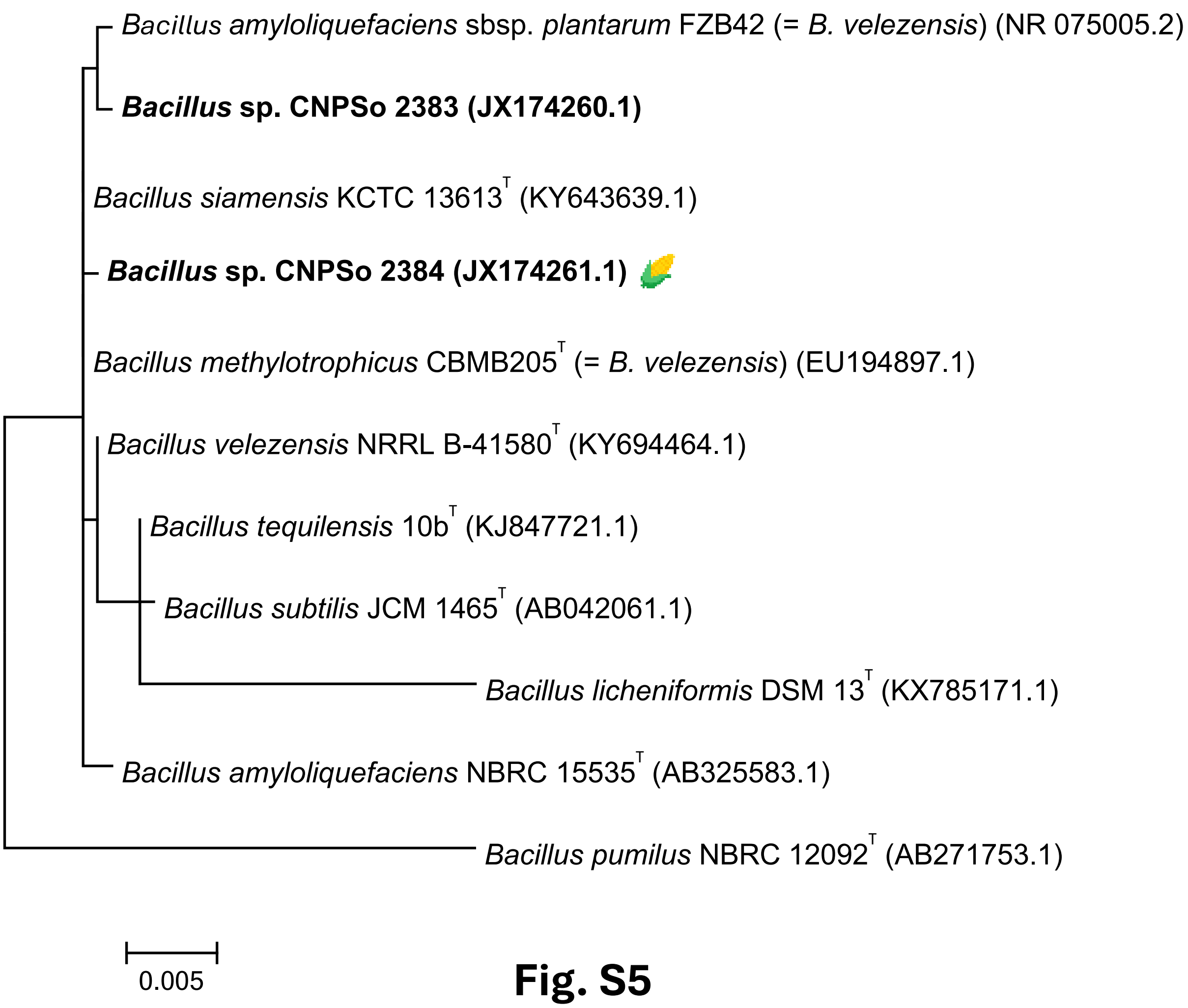
Bacillus amyloliquefaciens sbsp. plantarum FZB42 (= B. velezensis) (NR 075005.2)
Bacillus sp. CNPSo 2383 (JX174260.1)
Bacillus siamensis KCTC 13613T (KY643639.1)
Bacillus sp. CNPSo 2384 (JX174261.1)
Bacillus methylotrophicus CBMB205T (= B. velezensis) (EU194897.1)
Bacillus velezensis NRRL B-41580T (KY694464.1)
Bacillus tequilensis 10bT (KJ847721.1)
Bacillus subtilis JCM 1465T (AB042061.1)
Bacillus licheniformis DSM 13T (KX785171.1)
Bacillus amyloliquefaciens NBRC 15535T (AB325583.1)
Bacillus pumilus NBRC 12092T (AB271753.1)
0.005

Fig. S5
